# A DNA Methylation-Based Panel for the Prognosis and Diagnosis of Patients With Breast Cancer and Its Mechanisms

**DOI:** 10.3389/fmolb.2020.00118

**Published:** 2020-07-07

**Authors:** Xiao-Ping Liu, Jinxuan Hou, Chen Chen, Li Guan, Han-Kun Hu, Sheng Li

**Affiliations:** ^1^Department of Urology, Zhongnan Hospital of Wuhan University, Wuhan, China; ^2^Department of Thyroid and Breast Surgery, Zhongnan Hospital of Wuhan University, Wuhan, China; ^3^Department of Biological Repositories, Zhongnan Hospital of Wuhan University, Wuhan, China; ^4^Human Genetics Resource Preservation Center of Hubei Province, Wuhan, China; ^5^Department of Pharmacy, Zhongnan Hospital of Wuhan University, Wuhan, China

**Keywords:** breast cancer, DNA methylation, prognosis, diagnosis, immune landscape, genomic metrics

## Abstract

**Objective:**

To identify DNA methylation related biomarkers in patients with breast cancer (BC).

**Materials and Methods:**

A total of seven BC methylation studies including 1,438 BC patients or breast tissues were included in this study. An elastic net regularized Cox proportional hazards regression (CPH) model was used to build a multi-5′-C-phosphate-G-3′ methylation panel. The diagnosis and prognosis power of the panel was evaluated and validated using a Kaplan–Meier curve, univariate and multivariable CPH, subgroup analysis. A nomogram containing the panel was developed. The relationships between the panel-based methylation risk and the immune landscape and genomic metrics were investigated.

**Results:**

Sixty-eight CpG sites were significantly correlated with the overall survival (OS) of BC patients, and based on the result of penalized CPH, a 28-CpG site based multi CpG methylation panel was found. The prognosis and diagnosis role of the panel was validated in the discovery set, validation set, and six independent cohorts, which indicated that higher methylation risk was associated with poor OS, and the panel outperformed currently available biomarkers and remained an independent factor after adjusting for other clinical features. The methylation risk was negatively correlated with innated and adaptive immune cells, and positively correlated with total mutation load, SCNA, and MATH.

**Conclusions:**

We validated a multi CpG methylation panel that could independently predict the OS of BC patients. The Th2-mediated tumor promotion effect—suppression of innate and adaptive immunity—participated in the progression of high-risk BC. Patients with high methylation risk were associated with tumor heterogeneity and poor survival.

## Introduction

Breast cancer (BC) is still one of the most common causes of cancer-related deaths among women in the world (Harbeck and Gnant, 2017). With changes in lifestyle (obesity, radiation exposure, reduction of physical exercise, drinking, hormone replacement therapy during menopause, early age at first menstruation, and having children late or not at all) and the advancement of screening methods (self or clinical breast exams, mammography, genetic screening, ultrasonography, and imaging examination), the incidence of BC, in recent years, has become higher and higher (Ghoncheh et al., 2016; Wang et al., 2019). It was reported that nearly 1.7 million people were diagnosed with BC worldwide, which resulted in nearly half a million deaths (DeSantis et al., 2014; Ghoncheh et al., 2016; Molinie et al., 2017). For non-invasive and localized BC patients, surgical treatment is currently recognized as the standard of care, and surgical treatment can also be combined with systemic endocrine therapy, chemotherapy, and radiotherapy (Robert and Turner, 2019). Although the advancement in the screening and treatment options have made BC a chronic disease and significantly prolonged the survival of patients, BC is reported to have a recurrence rate of around 10% in early stage BC patients. For patients with metastatic BC that is considered an incurable disease with current existing treatment options, systematic salvage therapies (including chemotherapy, endocrine therapy, etc.) are recommended (Robert and Turner, 2019; Yang and Polley, 2019). However, long-term survival for this group of patients is dismal (less than 5%) (Vicini et al., 2018; Yang and Polley, 2019).

In recent years, multigene tests for the early diagnosis of BC have matured and emerged one after the other, which has greatly improved the situation for diagnosis and therapy of BC (Duffy et al., 2017; Colomer et al., 2018). However, there are still no well accepted diagnosis and prognosis markers for BC based on DNA methylation sites. In the present study, we tried to develop a multi-5′-C-phosphate-G-3′ (CpG) site based DNA methylation panel for the diagnosis and prognostication of patients with BC, and confirm its prognostic role in several independent cohorts.

## Materials and Methods

### Data Collection

In the present study, we included a total of seven BC methylation studies. The DNA methylation profile of The Cancer Genome Atlas Breast Invasive Carcinoma (TCGA-BRCA) (Cancer Genome Atlas, 2012; Ciriello et al., 2015), measured with Illumina Human Methylation 450 BeadChip, included a total of 890 BC samples and associated clinical information of BC patients and was downloaded from UCSC Xena^1^. A total of 62, 118, 188, 70, and 58 BC samples were included in GSE37754 (Putluri et al., 2014; Terunuma et al., 2014; Tang et al., 2018), GSE72245 (Jeschke et al., 2017), GSE75067 (Holm et al., 2016), GSE78754 (Mathe et al., 2016), and GSE72251 (Jeschke et al., 2017), respectively, and there were 40 normal breast samples and 80 BC samples in GSE66695^2^. Bisulfite converted DNA from the breast samples in the above datasets were hybridized to the Illumina Infinium 450k Human Methylation Beadchip. mRNA expression profile of the TCGA-BRCA including 1,217 samples and somatic variant data in “maf” format, were downloaded from GDC Data Portal^3^. The inclusion criteria of this study were as follows: the patients were newly diagnosed with BC, the patient’s survival information was well documented or the study contained both normal breast sample and BC samples, and the relevant BC patients received Illumina Human Methylation 450 BeadChip profiling.

### Preprocessing and Differentially Methylated CpG Site Analyses

The R package “ChAMP” was used to preprocess the beta-valued matrix of the TCGA-BRCA methylation data (Tian et al., 2017). Before the CpG methylation matrix was subjected to the ChAMP pipeline, CpG sites containing more than 90% of missing values in the matrix were deleted. Next, probes meeting the following criteria were filtered: (1) probes from X and Y chromosomes, (2) probes align to multiple locations, (3) probes in which the probed CpG falls near a SNP, (4) non-CpG probes, and (5) probes with less than three beads. Subsequently, the differentially methylated positions between BC samples and paired normal breast samples were identified through multiple linear models. Any probe that satisfied adj.pvalue < 0.05 and | deltaBeta | > 0.3 was considered significantly methylated.

### Identification of Multi-CpG Methylation Panel and Validation of Its Prognostication Value

Differentially methylated CpG sites were subjected to univariate Cox proportional hazards regression model (CPH) to screen CpG sites correlated with the overall survival (OS) of BC patients in the TCGA-BRCA. Then, the BC samples in the TCGA-BRCA cohort were randomly classified into a discovery set and validation set according to the ratio of 3:2. In the validation cohort, the OS-related CpGs were included in an elastic net regularized CPH model, which was optimized based on two hyperparameters (α and λ) tuned using 10-fold cross validation (Sill et al., 2014). Then, we performed feature selection according the elastic net regularized CPH, namely, CpGs with coefficients equaled to 0 were removed. Thus, the multi-CpG methylation panels were constructed based on the regularized CPH, and the associated risk scores of each BC patient in the discovery set, validation set, and other independent validation cohorts were derived based on the coefficients of the each CpG site in the CPH model and their corresponding beta values. Time-dependent receiver operator curve (ROC) at different time points (1-, 3-, 5-, 7-, 10-, and 15-years) were drawn to verify the prediction performance of the multi-CpG methylation panel using the R package “survivalROC.” Then, the optimal cutoff, obtained according to the results of time-dependent ROC, was used to classify patients into low-risk group and high-risk group. The Kaplan–Meier (KM) curve and univariate and multivariable CPH model were applied to characterize the prognostic role of the multi-CpG site panel. Moreover, BC patients in the discovery set and validation set were further divided into several subgroups, i.e., triple-negative BC (TNBC) versus non-triple negative BC (non-TNBC), early stage (stage I and stage II) BC versus advanced (stage III and stage IV) BC, young BC (BC patients younger than 65 years) versus old BC (BC patients older 65 years). Then, KM curves were drawn in these subgroups, respectively.

Finally, to further validate the prognostication value the multi-CpG methylation panel, we analyzed the correlations between the methylation levels of specific CpGs in the panel and associated mRNA expressions using Spearman’s correlation analysis in the discovery set and validation set, and BC samples were divided into two groups based on the cutoff estimated using the “surv_cutpoint” function in the R package “survminer,” and then, KM curves were also drawn to evaluate the survival differences between different risk groups.

### Clinical Application of the Multi-CpG Methylation Panel

In order to further clarify the application value of this multi-CpG methylation panel in clinical practice, we included it with other clinical phenotypes of BC patients in the discovery set (such as age and pathological stage) into a multivariable survival model, and based on this model, we drew a nomogram for clinicians to make decisions in the clinic. The performance of the nomogram was internally validated using 1,000 bootstraps and was calibrated at 3- and 5-years. Meanwhile, decision curve analysis (DCA; Vickers and Elkin, 2006) was perform to clarify the clinical benefit of the multi-CpG methylation.

### Comparison of the Multi-CpG Methylation Panel With Other Well-Known Signatures in Predictive Performance

Currently, several multi-CpG methylation panels including some DNA methylation and mRNA expression-based panels have been published. Therefore, based on the discovery set, validation set, and independent cohorts, we tried to compare the performance of the multi-CpG panel with the currently available prognostic signatures in terms of concordance index (C-index). The C-indexes of the signatures were calculated and compared using the R/Bioconductor package “survcomp” (Haibe-Kains et al., 2008; Schroder et al., 2011).

### Analyzing the Correlation Between the Multi-CpG Methylation Panel and Immune Infiltration

Thanks to the specific gene list of 24 immune cells provided by Bindea et al. (2013), we calculated the immune infiltration scores for specific immune cells based on the RNA-seq data in the TCGA-BRCA cohort using the ssGSEA method in the R package GSVA (Hanzelmann et al., 2013), and then the correlations between the multi-CpG methylation panel and infiltration score of specific immune cells were estimated using Spearman’s correlation. Meanwhile, differentially expressed genes between the multi-CpG methylation panel low-risk group and high-risk group were also identified using the R package “edgeR” (Dai et al., 2014), and the expression levels of genes from the 24 immune cell types between the groups were further compared using Wilcoxon rank sum test. Spearman’s correlation analysis was also performed between the multi-CpG methylation risk and cytolytic activity [calculated according to the formula suggested by Rooney et al. (2015)], overall immune cell and T cell infiltration scores [estimated as Senbabaoglu et al. (2016) suggested], the ratio between the expression of CD8^+^ T cell-specific genes versus Treg cell-specific gene, the ratio between the expression of immune stimulation genes versus immunosuppression genes, and the ratio between the expression of Th17 cell-specific genes versus Th2 specific genes.

### Analyzing the Correlations Between the Genomic Metrics and the Multi-CpG Methylation Panel Risk of Patients With BC

Somatic copy number alterations (SCNA) were known to drive tumorigenesis in a variety of human cancers including BC. Mutant-allele tumor heterogeneity (MATH; Mroz and Rocco, 2013) was considered a measure for the evaluation of intratumor genetic heterogeneity. Genomic mutations were generally considered to be an important driver of tumorigenesis and development. In the present study, the MATH score and total mutation of each BC patients in the discovery set and validation set were calculated using the R package “maftools” (Mayakonda et al., 2018), and then the correlations between the multi-CpG methylation panel and the MATH score, total mutation, and the SCNA derived from the publication of Davoli et al. (2017) were evaluated using Spearman’s correlation.

## Results

### Characteristics of BC Patients

According to the inclusion criteria, 786 samples in the TCGA-BRCA were included in the present study, 75 of which were normal breast tissues and the remaining 682 BC samples were used for subsequent model training and validation. A total of 460 BC patients were included in the discovery set, 198 of which were in the high-risk group (median age: 60 years) and 262 in the low-risk group (median age: 54 years) (Supplementary Table S1). A total of 305 BC patients were included in the validation set, 144 of which were in the high-risk group (median age: 62 years) and 161 in the low-risk group (median age: 55 years) (Supplementary Table S2). A total of 61 BC patients were included in GSE37754, 32 of which were in the high-risk group (median age: 57 years) and 29 in the low-risk group (median age: 46 years) (Supplementary Table S3). A total of 119 patients were included in GSE72251, 40 of which were in the high-risk group (median age: 59.3 years) and 79 in the low-risk group (median age: 57.6 years) (Supplementary Table S4). A total of 126 BC patients were included in GSE72245, eight of which were in the high-risk group (median age: 54.7 years) and 118 in the low-risk group (median age: 53.9 years) (Supplementary Table S5). A total of 181 BC patients were included in GSE75067, 52 of which were in the high-risk group (median age: 55.1 years) and 129 in the low-risk group (median age: 47.2 years) (Supplementary Table S6). A total of 66 BC patients were included in GSE78754, 21 of which were in the high-risk group (median age: 61.0 years) and 45 in the low-risk group (median age: 47.0 years). Furthermore, there were 80 BC patients and 40 normal controls in GSE66695. Detailed characteristics of BC patients can be found in the Supplementary Tables.

### Development of the Multi-CpG Methylation Panel

**FIGURE 1:**
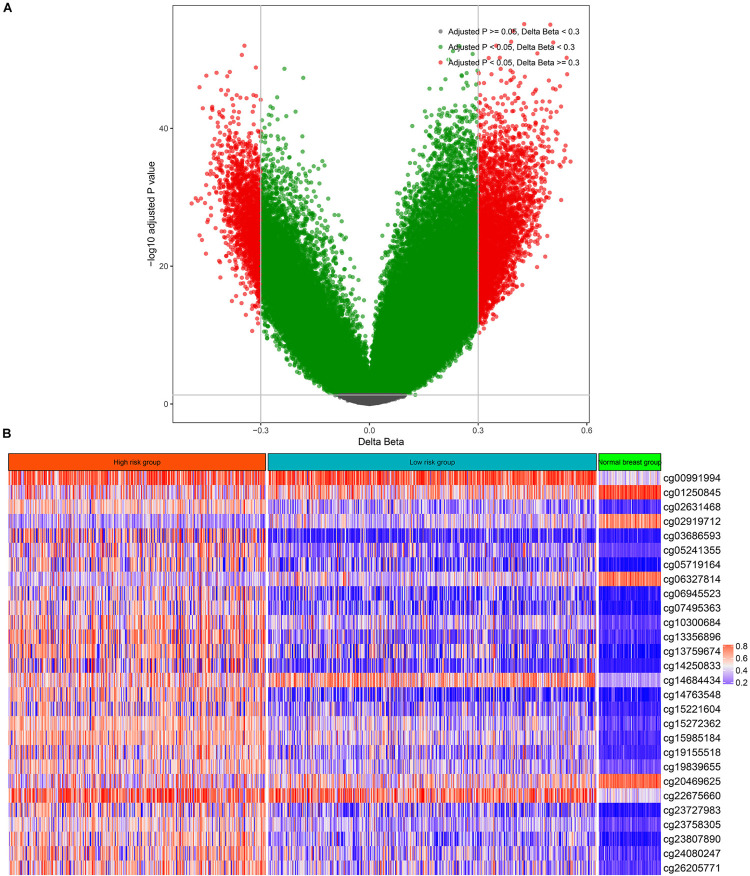
Differentially methylated CpG sites between breast cancer and normal breast tissue **(A)** and the methylation levels of the 28 CpG sites in the high-risk group, low-risk group, and normal breast tissues **(B)**.

There were 75 pairs of normal breast tissue and BC tissue in the TCGA-BRCA cohort, thus, we calculated the differentially methylated CpGs sites between normal BC and BC tissues. According to the above screening criteria (adjusted *p* value < 0.05 and | deltaBeta | > 0.3), a total of 5,736 differentially methylated CpG sites were found between normal breast tissue and BC (Figure 1A). The above differentially methylated CpG sites were subjected to the univariate CPH model to further screen for methylation sites that were significantly correlated with the OS of BC patients. Consequently, a total of 68 CpG sites that significantly (*p* < 0.005) correlated with the OS of BC patients (Supplementary Table S7) were selected as candidates for the development of the multi-CpG methylation panel. After 10-fold cross validation, a pair of optimal hyperparameters were found (α = 0.0041, λ = 2.8244, Supplementary Figure S1). As a result of the optimal elastic penalized.

CPH model, 28 methylated CpG sites whose coefficients were not equal to zero in the model were found and utilized to build the multi-CpG methylation panel based on the methylation levels of these CpG sites (Figure 1B and Supplementary Table S8) and their corresponding coefficients in the model.

### Evaluation and Validation of the Prognostication Ability of the Multi-CpG Methylation Panel

**FIGURE 2:**
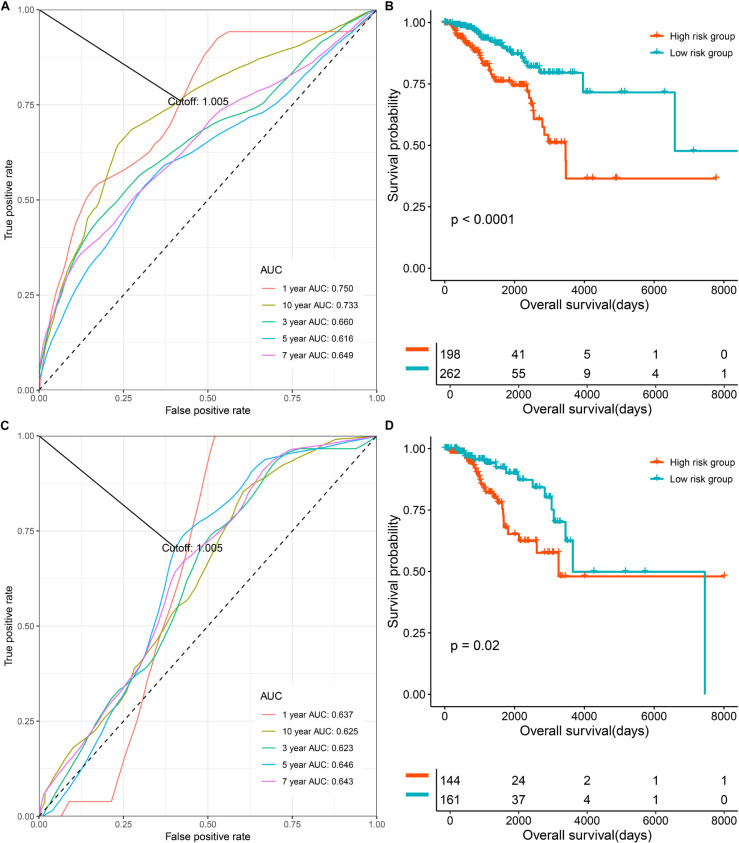
The prognostic role the multi CpG methylation panel in the discovery set and validation set. **(A)** Time-dependent ROC analysis on the overall survival of patients in the discovery set. **(B)** Kaplan–Meier curve on the overall survival of patients with low methylation risk versus high methylation risk in the discovery set. **(C)** Time-dependent ROC analysis on the overall survival of patients in the validation set. **(D)** Kaplan-Meier curve on the overall survival of patients with low methylation risk versus high methylation risk in the discovery set.

To evaluate and validate the prognostication ability of the multi-CpG methylation panel, we performed time-dependent ROC analysis, KM analysis, and a univariate and multivariable CPH model. As shown in Figure 2, the results of the time-dependent ROC analysis suggested that areas under the curve (AUCs) at 1-, 3-, 5-, 7-, and 10-year for the prediction of the OS of BC patients in the discovery set were 0.75, 0.66, 0.616, 0.649, and 0.733, and according to the optimal cutoff 1.005 (Figure 2A and Supplementary Figure S2A), BC patients were classified into two risk groups, which have significantly different OSs (*p* < 0.0001, Figure 2B). In the validation set, the multi-CpG methylation panel was also good enough to predict the OS of BC patients (AUCs at 1-, 3-, 5-, 7-, and 10-year were 0.637, 0.623, 0.646, 0.643, and 0.625, respectively, Figure 2C and Supplementary Figure S2B) and the patients were classified into significantly different risk groups by the methylation score (*p* = 0.02, Figure 2D).

Subgroup analysis indicated that a lower risk of methylation was, or, tended to be associated with better prognosis of patients with TNBC and non-TNBC in the discovery set (Supplementary Figures S3A,B) and validation set (Supplementary Figures S3C,D). The results of subgroup analysis, based on pathological stage, suggested that the multi-CpG methylation panel can stratify patients into significantly different survival groups in the discovery set and validation set (Supplementary Figures S3E–H), and although the survival difference of patients with early (stage I and stage II) BC in the two risk groups was not statistically significant, patients in the low-risk group were obviously related with better survival compared with those in the high-risk group for more than 10 years (Supplementary Figure S3G). Similar trends could also be found in different age groups (patients who were or not younger than 65 years) (Supplementary Figures S3I–L).

In addition, the prognostic role of the multi-CpG methylation panel was also evaluated in several independent cohorts. The time- dependent ROC analyses and KM curves also suggested that the multi-CpG methylation panel showed good performance in predicting the OS of patients and was able to significantly divide patients of these cohorts into different survival groups (Supplementary Figure S4).

Finally, the univariate and multivariable CPH model suggested that the multi-CpG methylation panel was an independent prognostic factor in BC patients (Supplementary Tables S9–S15). Furthermore, the methylation risk in BC samples was significantly higher compared with that in the normal breast tissues (*p* < 0.0001, AUC = 0.97, Supplementary Figure S5), indicating that it could be selected as a candidate for the screening or diagnosis of BC.

### Associations Between CpG Methylation and Corresponding mRNA Expression

We further analyzed the correlations between the methylation levels of the 28 CpG sites and their regulated mRNA expression levels. Given that five genes (C3orf26, DEPDC6, FAM38B, C7orf53, and WDR69) regulated by the CpG sites were not found in the mRNA expression profile, we could only analyze the associations between the remaining CpG methylation and mRNA expression. As shown in Supplementary Figure S6, cg07495363 and cg13356896 were found to be negatively correlated with BOLL expression (*R* = -0.17, and *R* = -0.092, respectively). cg14684434 methylation was negatively correlated with the expression of SNX18 (*R* = -0.19), cg01250845 methylation level was negatively correlated with the expression of RAB30, cg19839655 methylation was negatively correlated with the expression of CD40 (*R* = -0.39), cg23727983 methylation was negatively correlated with the expression of DDX25 (*R* = -0.27), cg15221604 methylation level was negatively correlated with ROBO3 expression (*R* = -0.12), and cg10300684 methylation was negatively correlated with the expression of FOXG1 (*R* = -0.22). Furthermore, cg19155518 methylation was positively correlated with the expression of GRIK2 (*R* = 0.2), cg13759674 methylation was positively correlated with the expression level of GRIN1 (*R* = 0.096), cg02631468, cg14763548, and cg15272362 methylation levels were positively correlated with the expression of VSX1 (*R* values were equal to 0.12, 0.087, and 0.071, respectively), cg05719164 methylation was positively correlated with the expression of LHX4 (*R* = 0.12), cg26205771 methylation was positively correlated with the expression of NPBWR1 (*R* = 0.11), cg20469625 methylation was positively correlated with the expression of ZFPM2 (*R* = 0.13), cg05241355 methylation was positively correlated with the expression of OTX2 (*R* = 0.13), and cg24080247 methylation was positively correlated with the expression level of SIM2 (*R* = 0.5). However, cg23758305, cg15985184, cg22675660, cg06945523, and cg14250833 were not significantly correlated with their regulated mRNA expressions. Although not all corresponding mRNAs could stratify BC patients into different survival groups, the combination of these mRNAs showed good performance in predicting the OS of patients with BC (the ROC curve in Supplementary Figure S6), and it could significantly stratify patients into different survival groups (the last survival curve in Supplementary Figure S6).

### Development and Validation of the CpG Methylation Panel Containing Nomogram for the Prediction of the Survival of BC Patients

**FIGURE 3:**
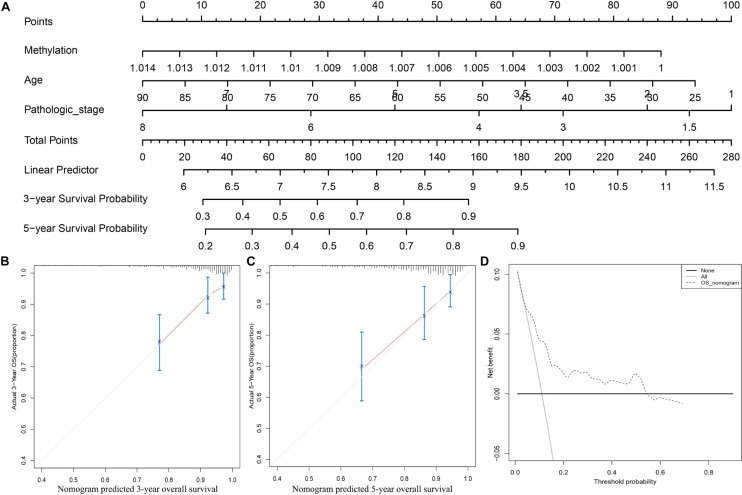
Clinical application of the multi CpG methylation panel. **(A)** The multi CpG methylation panel containing nomogram; **(B)** Calibration analysis of the nomogram for the prediction of 3-year overall survival; **(C)** Calibration analysis of the nomogram for the prediction of 5-year overall survival; **(D)** Decision curve analysis to identify the clinical usability of the nomogram. The pathological stage has been coded according to following criteria: Stage IA = 1, Stage IB = 2, Stage I = 1.5, Stage IIA = 3, Stage IIB = 4, Stage II = 3.5; Stage IIIA = 5, Stage IIIB = 6, Stage IIIC = 7, Stage III = 6, Stage IV = 8.

To further apply the CpG methylation panel into clinical application, we incorporated the multi-CpG methylation panel together with age and pathological stage into a multivariable survival model, and then a nomogram was drawn as shown in Figure 3A. In clinical practice, a physician could predict the probabilities of 3- and 5-year OS of patients based on the nomogram. To predict one’s 3- and 5-year probabilities, the physician could draw vertical lines to the “Points” line to estimate corresponding points related with the methylation risk, age, and pathological stage of a patients, and then the probabilities of 3- and 5-year OS of the patients can be estimated based on the value of “Total Points,” which is the sum of the respective points of the above clinical phenotypes. Internal validation with bootstrap suggested that the nomogram remained a powerful predictor (C-index = 0.7714). As shown in Figures 3B,C, calibration analysis results showed that the predicted 3- and 5-year survival probabilities were in good agreement with the actual 3- and 5-year survival probabilities. Meanwhile, the results of DCA suggested that the multi methylation panel containing nomogram showed better clinical benefit with a threshold probability ranging from 10 to 60%, indicating that the nomogram has a very good application prospect in clinical settings (Figure 3D).

### Comparison of the Predictive Power of the Multi-CpG Methylation Panel With Other Multi-Molecular Biomarkers in BC

**FIGURE 4:**
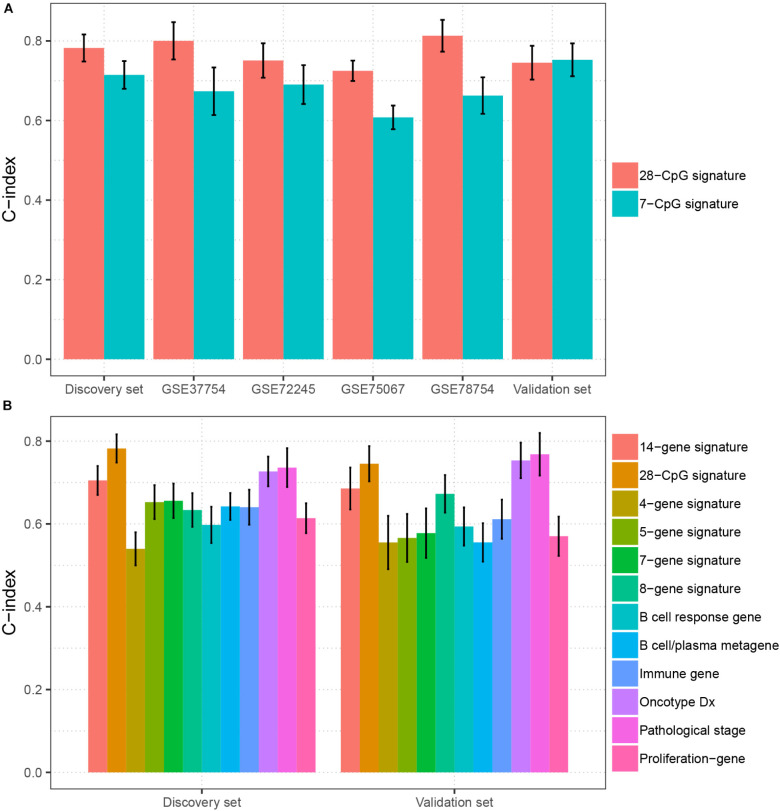
Comparison of the prediction performance between the multi CpG methylation panel and multi molecular biomarkers. **(A)** The multi CpG methylation panel (28-CpG methylation) versus the 7-CpG methylation signature in the discovery set, validation set, and independent cohorts. **(B)** The multi-CpG methylation panel versus 10 mRNA based multigene signature and the pathological stage in the discovery set and validation set.

To evaluate the clinical potential of the multi CpG methylation panel, we compared it with several currently existing multi molecular biomarkers [7-CpG signature (Tao et al., 2019), 8-gene signature (Cui et al., 2019), 4-gene signature (Qi et al., 2019), 7-gene signature (Teschendorff and Caldas, 2008), 14-gene signature (Yau et al., 2010), 5-gene signature (Yau et al., 2013), B cell/plasma metagene (Bianchini et al., 2010), proliferation gene (Oh et al., 2012), immune gene (Oh et al., 2012), B cell response gene (Ascierto et al., 2012), Oncotype DX (Toi et al., 2010), and pathology stage (Runowicz et al., 2016)] in BC. As shown in Figure 4A, our multi CpG methylation panel outperformed the 7-CpG signature in the discovery set, GSE78754, GSE75067, GSE72245, and GSE37754, and was comparable with the 7-CpG signature in the validation set. Furthermore, our multi CpG methylation panel was superior to the other 11 multigene signatures (8-gene signature, 4-gene signature, 7-gene signature, 14-gene signature, 5-gene signature, B cell/plasma metagene, proliferation gene, immune gene, B cell response gene, Oncotype DX, and pathology stage) in the discovery set and it was also superior to the other nine multigene signatures and was comparable to the Oncotype DX and the pathological stage (Figure 4B). The above findings suggest that the multi CpG methylation panel has excellent predictive performance and has good application value in clinical settings.

### The Association Between the Multi-CpG Methylation Panel and the Immune Landscape of BC Patients

**FIGURE 5:**
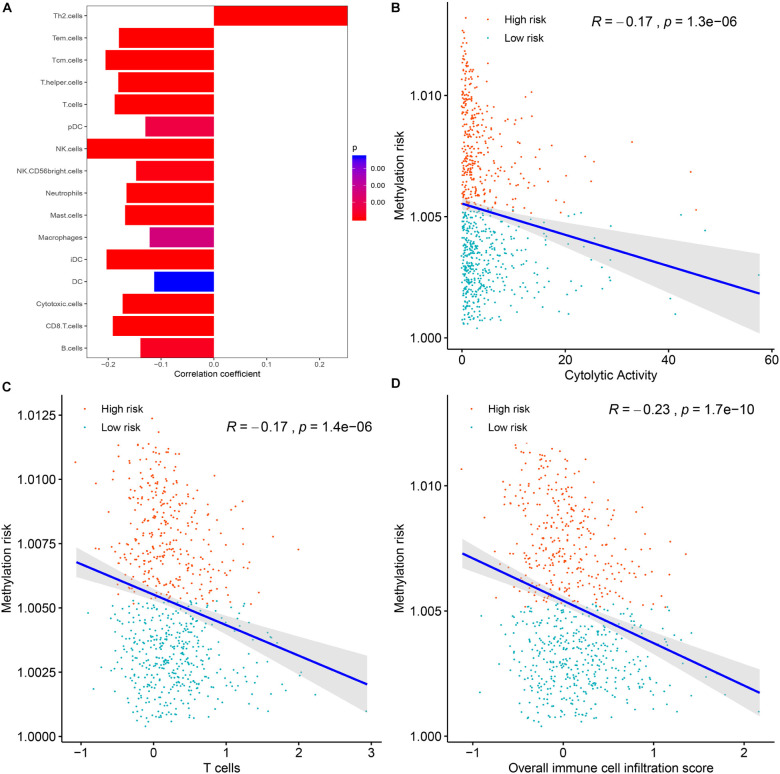
The correlations between the methylation risk and the immune infiltration of BC patients. **(A)** The correlations between the methylation risk and the Th2 cells, Tem cells, Tcm cells, T helper cells, T cells, pDC, NK cells, NK CD56 bright cells, neutrophils, mast cell, iDCs, DCs, cytotoxic cells, CD8 + T cell, and B cells. **(B–D)** The correlations between the methylation risk and the cytolytic activity, the overall immune infiltration score, and the overall T cell infiltration score.

**FIGURE 6:**
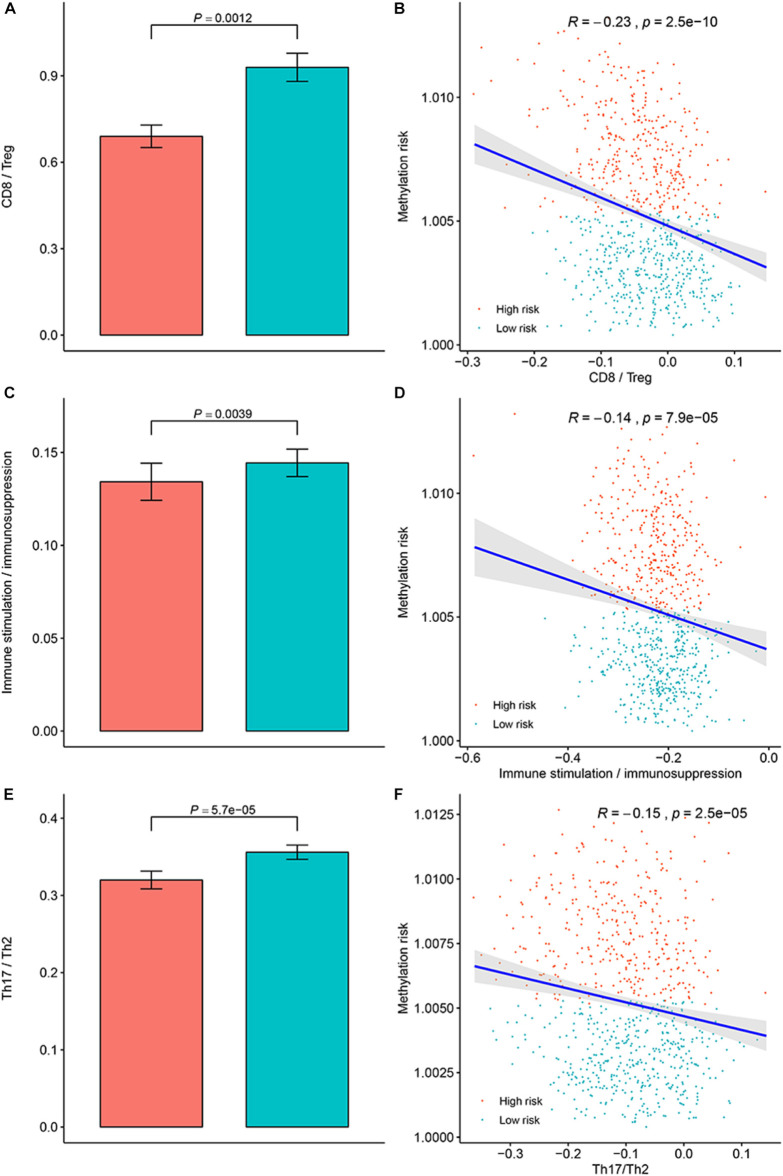
Evaluation of the distribution of the ratios between protumorigenic immune cells or immune stimulation molecular versus antitumorigenic cells or immune suppression in the methylation low-risk group and methylation high-risk group. **(A)** Ratio between CD8^+^ cell versus Treg cell in different methylation risk groups; **(B)** Correlation between the methylation risk and ratio between CD8^+^ cell versus Treg cell in different methylation risk groups. **(C)** Ratio between immune stimulation molecular versus immune suppression molecular in different methylation risk groups; **(D)** Correlation between the methylation risk and the ratio between immune stimulation molecular versus immune suppression molecular. **(E)** Ratio between Th17 cell versus Th2 cell in different methylation risk groups. **(F)** Correlation between the methylation risk and ratio between Th17 cell versus Th2 cell. Notes: the blue bar represent the low-risk group, while the red bar represent the high-risk group.

To clarify the specific mechanism of the effect of the multi-CpG methylation panel on the survival of BC patients, we tried to analyze the association between the risk of the methylation and the immune infiltration of BC patients, which was calculated based on the ssGSEA method. As shown in Figure 5A, results of Spearman’s correlation suggested that the methylation risk was positively correlated with Th2 cells, while it was negatively correlated with other immune stimulation cells [including Tem cells (effector memory T cells), Tcm cells (central memory T cells), T helper cells, T cells, pDC (Plasmacytoid dendritic cell), NK cells, NK CD56 bright cells, neutrophils, mast cells, iDCs (immature dendritic cells), DCs, cytotoxic cells, CD8^+^ T cell, and B cells]. Meanwhile, genes specific for B cells (BLK, CD19, COCH, CR2, FCRL2, IGHA1, MS4A1, and TCL1A), CD8^+^ T cells (CD8B and GZMM), cytotoxic cells (CTSW, KLRB1, and ZBTB16), DCs (CCL17, HSD11B1, and NPR1) eosinophils (TKTL1), iDCs (CD1A, CD1B, CD1C, CD1E, CH25H, CLEC10A, FABP4, and MMP12), macrophages (CHIT1, DNASE2B, PTGDS, SCG5, and SULT1C2), mast cells (CALB2, CMA1, CTSG, GATA2, HDC, HPGD, NR0B1, SCG2, and TPSB2), neutrophils (FCGR3B, and G0S2) NK CD56bright cells (RRAD, and XCL1), NK cells (IGFBP5, PDLIM4, and XCL1), T cells (CD3D, CD3E, ITM2A, SH2D1A, TRAC, and TRAT1),T helper cells (ITM2A), Tcm cells (CDC14A), Tfh cells (B3GAT1, CXCL13, and PVALB), Th1 cells (APOD, and GGT1), and Th2 cells (ANK1, BIRC5, CENPF, and NEIL3) were differently expressed between the multi CpG methylation low-risk group and the multi CpG methylation high-risk group (Supplementary Figure S7). Cytolytic activity represented an important indicator of anti-tumor response, thus, Spearman’s correlation was conducted, and its result suggested that higher methylation risk was associated with lower cytolytic activity (Figure 5B). Spearman’s rank correlation analysis revealed weak negative associations between the methylation risk and TIS and OIIS (Figures 5C,D). The ratio between protumorigenic immune cells or immune stimulation molecular versus antitumorigenic cells or immune suppression is more likely to determine whether the net effect of these cells is tumor promotion versus inhibition, compared with the absolute count of certain immune cell types. As shown in Figure 6, the ratio between CD8^+^ T cells versus Treg cells (*P*_*wilcox.test*_ = 0.0012, *R*_*spearman*_ = −0.23), the ratio between immune stimulation molecular (IFN-γ,IL-1A, IL-1B, and IL-2) versus immune suppression molecular (IL-4, IL-10, IL-11, and TGFB1) (*P*_*wilcox.test*_ = 0.0039, *R*_*spearman*_ = −0.14), and the ratio between Th17 cells versus Th2 cells (*P*_*wilcox.test*_ < 0.0001, *R*_*spearman*_ = −0.15) were significantly different between the two risk groups and negatively correlated with the methylation risk of BC patients.

### The Associations Between the Multi-CpG Methylation Panel and the Genomic Metrics of BC Patients

**FIGURE 7:**
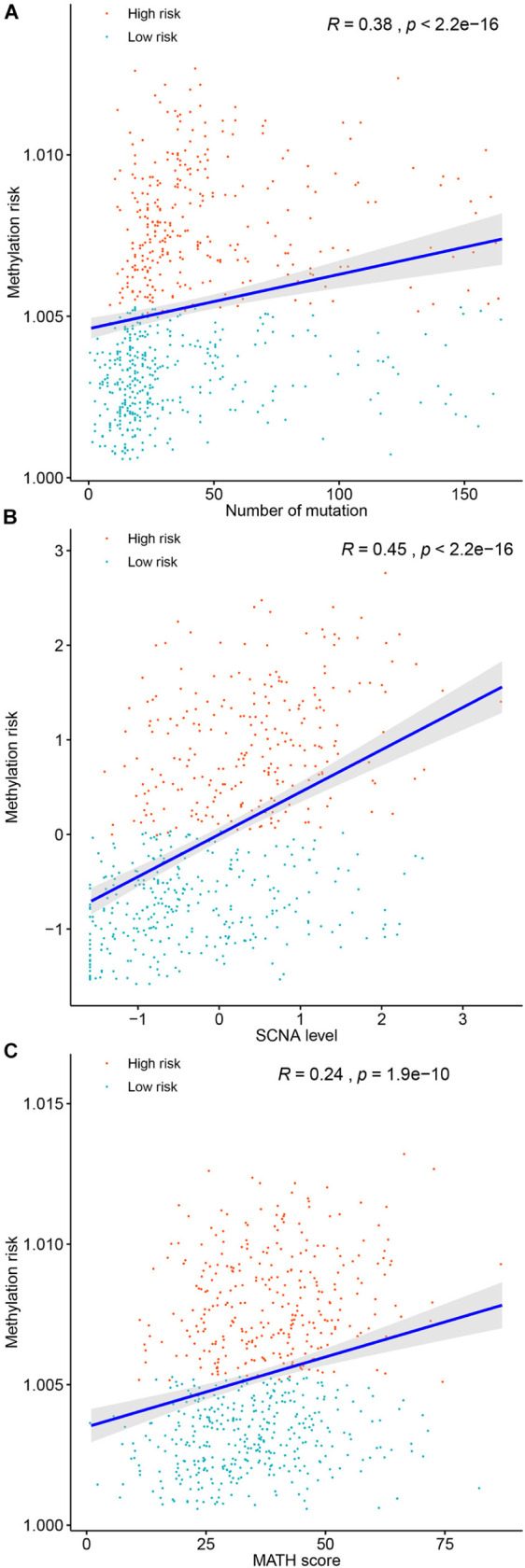
Correlations between the methylation risk and the total mutation **(A)**, SCNA level **(B)**, and MATH score **(C)**.

Genomic metrics including (total mutation load, SCNA, and MATH) were reported in multiple cancers and were demonstrated to be associated the progression of serval human cancers. Therefore, we investigated the associations between total mutation load, SCNA, MATH, and the DNA methylation risk of BC patients. As shown in Figure 7, higher methylation risk of BC patients were significantly associated with higher total mutation number (*R* = 0.38, *p* < 0.0001, Figure 7A), higher SCNA (*R* = 0.45, *p* < 0.0001, Figure 7B), higher MATH (*R* = 0.24, *p* < 0.0001, Figure 7C).

## Discussion

As mentioned above, although current screening, diagnosis, and treatment of BC have been improved significantly, the clinical prognosis of some BC patients remains very poor. Therefore, it is of great significance to develop new molecular markers based on different detection methods. In the present study, we first identified differentially methylated CpG sites between BCs and normal breast tissues, and then screened survival related CpG sites using the univariate CPH model. Based on the survival related CpG sites, an elastic net penalized CPH model was trained and optimized using 10-fold cross validation, which resulted in a 28-CpG-site based methylation panel. The multi CpG methylation panel was demonstrated to be associated with the OS of patients with BC, namely, higher multi CpG methylation panel based methylation risk was associated with worse OS of BC patients, and it remained to be an independent prognostic factor after adjusting for other clinical characteristics of BC patients. Subgroup analysis showed that the multi CpG methylation panel was still able to divide BC patients into different survival groups in different subgroups (TNBC versus non-TNBC, early stage BC versus late stage BC, and patients younger than 65 years versus patients older than 65 years). Internal validation suggested the multi CpG methylation panel remained a powerful predictor. Finally, when comparing it with other multi molecular biomarkers, the multi CpG methylation panel showed excellent performance. Altogether, we identified an independent biomarker for the diagnosis and prognosis of patients with BC.

The tumor microenvironment is of great significance to the occurrence and development of tumors and the clinical manifestations and prognosis of patients. At the same time, tumor immune cell infiltration is an important part of the tumor microenvironment, which has been applied to categorize multiple human cancers into different immune groups with diverse heterogeneity and survival (Thorsson et al., 2018). Therefore, analysis of the relationship between methylation risk and immune cell composition in BC patients would contribute to understanding the patient’s immune status, the relationship between genomic methylation and immune infiltration, and potential immunotherapy. In the present study, we found that the methylation risk of BC patients was positively correlated with Th2 cells, while higher methylation risk was correlated with lower infiltration of Tem cells, Tcm cells, T helper cells, T cells, pDC, NK cells, NK CD56 bright cells, neutrophils, mast cell, iDCs, DCs, cytotoxic cells, CD8^+^ T cell, and B cells. The Th2-mediated immune response had long been recognized as a favorable factor for tumor proliferation by promoting fibroblast thymic stromal lymphopoietin production, angiogenesis, and by suppressing the cell-mediated immune response (Ellyard et al., 2007; De Monte et al., 2011). However, T cells, DCs, NK cells, neutrophils, mast cells, and BC cells were conventionally considered to be an immunologic defense for anti-tumor activity. The ratio between protumorigenic immune cells or immune stimulation molecular versus antitumorigenic cells or immune suppression is more likely to determine whether the net effect of these cells is tumor promotion versus inhibition, compared with the absolute count of certain immune cell types. As shown in Figure 6, the ratios between protumorigenic immune cells or immune stimulation molecular versus antitumorigenic cells, or immune suppression were significantly increased in patients with higher methylation risk. Driven by neoepitopes, cytolytic activity was demonstrated to be related to counter-regulatory immune responses and improved survival of patients. Our study suggested that higher methylation risk was associated with poor cytolytic activity. Thus, it could be summarized that the Th2-mediated tumor promotion effect participated in the progression of BC, and innate and adaptive immunity in high-risk patients were suppressed.

Relationships between genomic metrics (total mutation, SCNA or Tumor aneuploidy, and MATH) and tumor heterogeneity and survival of tumor patients, have become new hot spots and trends in the field of tumor research in recent years. We identified that lower methylation risk was correlated with lower total mutation load, which resulted in better prognosis of BC patients and was consistent with previous observations in other tumors (Li et al., 2018; Samstein et al., 2019). Somatic copy number alterations, or aneuploidy, is widely presented in human cancers and has been considered a driving force in carcinogenesis (Davoli et al., 2017). Somatic copy number alterations is well known for its ability to promote tumor cell proliferation and leads to poorer survival of cancer patients (Davoli et al., 2017). This is similar to our findings that a higher methylation risk in patients is related to higher SCNA levels, and in return they lived for a shorter period than others. One of the measures of intratumor heterogeneity, namely a higher MATH score, represented a higher clonal and genetic heterogeneity which promoted tumor progression (McGranahan and Swanton, 2017). Our study suggested that a higher MATH score in patients, was significantly related with a higher methylation risk, and patients then had a poor prognosis compared with others.

Although DNA methylation is known to be a negative regulator of mRNA expression, in our study, we had different findings (Anastasiadi et al., 2018). Except for the five CpGs that had no corresponding mRNAs in the mRNA expression profile of the TCGA-BRCA cohort, eight CpG sites (cg07495363, cg13356896, cg14684434, cg01250845, cg19839655, cg23727983, cg15221604, and cg10300684) were negatively correlated with their corresponding mRNAs, and ten CpG sites (cg19155518, cg13759674, cg02631468, cg14763548, cg15272362, cg05719164, cg26205771, cg20469625, cg05241355, and cg24080247) were found to be positively correlated with their target genes, however, there were still five CpG sites (cg23758305, cg15985184, cg22675660, cg06945523, and cg14250833) that had no significant correlation with their target genes. This was similar to the conclusions of Spainhour et al. (2019). They analyzed the relationship between DNA methylation and corresponding mRNA expression using a variety of human tumors in TCGA. The results showed that nearly 30% of methylation sites were positively correlated with their corresponding mRNAs, and the correlation patterns were tissue dependent (Spainhour et al., 2019). Thus, the role of DNA methylation on the mRNA expression remains to be further elucidated. Although not all genes regulated by their DNA methylation could stratify BC patients into significantly different survival groups, the combination of these mRNAs could significantly classify BC patients into different risk groups (Supplementary Figure S6), which further confirmed the predictive performance of the multi CpG methylation panel for BC patients.

There were several limitations in our study. First, not all survival differences between the low-risk group and high-risk group were statistically significant in the different subgroups. For example, although the OS of patients with TNBC in the low-risk group tended to be better than that in the high-risk group; the difference did not reach statistical significance (*p* = 0.73, Supplementary Figure S3C). This might be due to the small number of patients in both groups (only 15 in the high-risk group and 14 in the low-risk group). Similar situations were found in patients with early stage BC in the validation set and patients older than 65 years in the validation set. Therefore, the conclusions should be interpreted with caution, and more patients are needed to verify them. Second, this study was a retrospective study, thus, large scale, prospective studies are also needed to further validate the conclusions. Third, although seven BC methylation studies retrieved from GEO and TCGA were included in the present study, the reporting format of the clinical characteristics of patients in these studies was not completely consistent. For example, in GSE37754, the original authors reported age, node status, neoadjuvant therapy, hormone therapy, and chemotherapy, while the original authors of GSE72251 reported age, subtype, grade, size, node status, and hormone receptors. More homogeneous studies are required to further validate the conclusion of the study. Finally, given that all the independent cohorts did not discuss the treatments of BC patients in the original studies, we could not evaluate the effect of treatment on the prognosis of BC patients, thus, future research should take into account the patient’s treatment.

In conclusion, we identified and validated a 28-CpG based multi CpG methylation panel that could classify BC patients into significantly different survival groups, and it remained to be an independent prognostic factor after adjusting for other clinical characteristic of BC patients. The Th2-mediated tumor promotion effect participated in the progression of high-risk BC, and innate and adaptive immunity in high-risk patients were suppressed. The multi CpG methylation panel-based methylation risk was associated with tumor heterogeneity and survival of BC patients.

## Data Availability Statement

The datasets generated for this study can be found in the TCGA-BRCA from https://xenabrowser.net/datapages/. GSE37754, GSE72245, GSE75067, GSE78754, and GSE72251 from https://www.ncbi.nlm.nih.gov/geo/.

## Author Contributions

SL designed the study. X-PL collected the data, performed the statistical analysis, conducted the methylation detection, and wrote the manuscript. JH participated in the methylation detection. CC, LG, H-KH reviewed the manuscript. All authors contributed to the article and approved the submitted version.

## Conflict of Interest

The authors declare that the research was conducted in the absence of any commercial or financial relationships that could be construed as a potential conflict of interest.

## Footnotes

1 https://xenabrowser.net/datapages/

2 https://www.ncbi.nlm.nih.gov/gds/?term=GSE66695

3 https://portal.gdc.cancer.gov

## Supplementary Material

FIGURE S1 Optimal hyperparameters (α and λ) for the elastic net penalized Cox proportional hazards regression model.

FIGURE S2 The prognostic role the multi CpG methylation panel in the discovery set and validation set. **(A)** Time-dependent ROC analysis on the overall survival of patients in the discovery set. **(B)** Time-dependent ROC analysis on the overall survival of patients in the validation set.

FIGURE S3 Kaplan-Meier curve analysis on the overall survival in different subgroups. **(A)** Triple negative breast cancer in the discovery set; **(B)** Non-triple negative breast cancer in the validation set; **(C)** Triple negative breast cancer in the discovery set; **(D)** Non-triple negative breast cancer in the validation set; **(E)** Early stage breast cancer in the discovery set; **(F)** Advanced stage BC in the discovery set. **(G)** Early stage breast cancer in the validation set; **(H)** Advanced stage BC in the validation set. **(I)** Patients younger than 65 years in the discovery set; **(J)** Patients older than 65 years in the discovery set; **(K)** Patients younger than 65 years in the validation set; **(L)** Patients older than 65 years in the validation set.

FIGURE S4 The prognostic value of the multi CpG methylation panel in five independent validation cohort. **(A)** Time- dependent ROC analysis for predicting the OS of patients in GSE37754. **(B)** KM curves on the OS of patients in GSE37754. **(C)** Time- dependent ROC analysis for predicting the OS of patients in GSE72245. **(D)** KM curves on the OS of patients in GSE72245. **(E)** Time-dependent ROC analysis for predicting the OS of patients in GSE72251. **(F)** KM curves on the OS of patients in GSE72251. **(G)**Time- dependent ROC analysis for predicting the OS of patients in GSE75067. **(H)** KM curves on the OS of patients in GSE75067. **(I)** Time- dependent ROC analysis for predicting the OS of patients in GSE78754. **(J)** KM curves on the OS of patients in GSE78754.

FIGURE S5 The diagnostic performance of the multi CpG methylation panel in GSE66695. **(A)** The methylation risk of breast cancer patients and normal controls. **(B)** ROC curve for the classification of the multi CpG methylation panel.

FIGURE S6 The correlations between the methylation levels of CpG sites and their corresponding mRNAs and the survival relevance of these mRNA on the overall survival breast cancer patients.

FIGURE S7 The expression levels of genes specific for immune cells and differently expressed between the low-risk group and high-risk group. Notes: genes for B cells:BLK, CD19, COCH, CR2, FCRL2, IGHA1, MS4A1, and TCL1A; Genes for CD8^+^ T cells: CD8B and GZMM; genes for cytotoxic cells: CTSW, KLRB1, and ZBTB16; genes for DCs: CCL17, HSD11B1, and NPR1; gene for eosinophils: TKTL1; genes for iDCs: CD1A, CD1B, CD1C, CD1E, CH25H, CLEC10A, FABP4, and MMP12; genes for macrophages: CHIT1, DNASE2B, PTGDS, SCG5, and SULT1C2; genes for mast cells: CALB2, CMA1, CTSG, GATA2, HDC, HPGD, NR0B1, SCG2, and TPSB2; genes for neutrophils: FCGR3B, and G0S2; genes for NK CD56bright cells: RRAD, and XCL1; genes for NK cells: IGFBP5, PDLIM4, and XCL1; genes for T cells CD3D, CD3E, ITM2A, SH2D1A, TRAC, and TRAT1; gene for T helper cells: ITM2A; gene for Tcm cells: CDC14A; genes for Tfh cells: B3GAT1, CXCL13, and PVALB; genes for Th1 cells: APOD, and GGT1; genes for Th2 cells: ANK1, BIRC5, CENPF, and NEIL3.
