# Erratum: A DNA Methylation-Based Panel for the Prognosis and Diagnosis of Patients With Breast Cancer and Its Mechanisms

**DOI:** 10.3389/fmolb.2020.596445

**Published:** 2020-11-12

**Authors:** Frontiers Production Office

**Affiliations:** Frontiers Media SA, Lausanne, Switzerland

**Keywords:** breast cancer, DNA methylation, prognosis, diagnosis, immune landscape, genomic metrics

Due to a production error, the word “diagnosis,” in the title, was written incorrectly as “dagnosis.”

The correct title is: “A DNA Methylation-Based Panel for the Prognosis and Diagnosis of Patients With Breast Cancer and Its Mechanisms.”

The publisher apologizes for this mistake. The original article has been updated.

