# Supplementary material for: A DNA Methylation-Based Panel for the Prognosis and Diagnosis of Patients With Breast Cancer and Its Mechanisms
Source: Front Mol Biosci. 2020 Jul 7;7:118. doi: 10.3389/fmolb.2020.00118 (PMC7358612; doi:10.3389/fmolb.2020.00118)
Supplement: FIGURE S1 — Optimal hyperparameters (α and λ) for the elastic net penalized Cox proportional hazards regression model. [file Data_Sheet_1.docx]

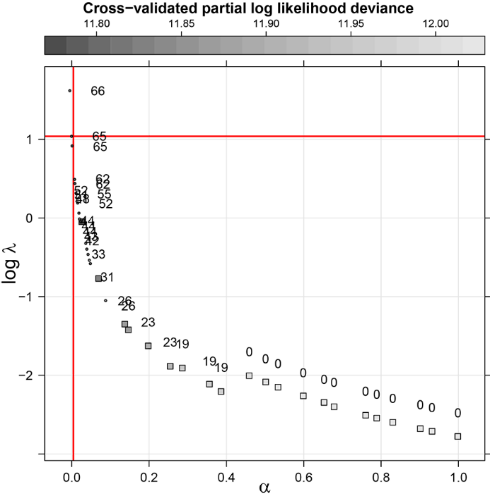


**Supplementary figure 1** Optimal hyperparameters (α and λ) for the elastic net penalized Cox proportional hazards regression model.


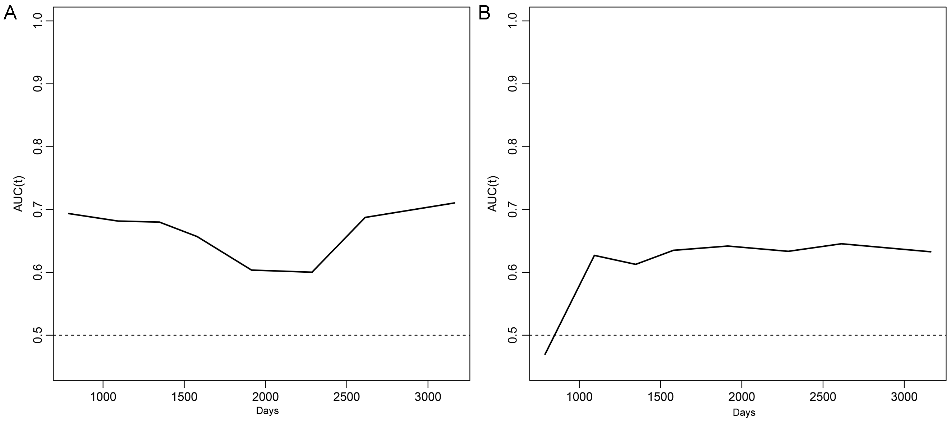


**Supplementary figure 2** The prognostic role the multi CpG methylation panel in the discovery set and validation set. (A) Time-dependent ROC analysis on the overall survival of patients in the discovery set. (B) Time-dependent ROC analysis on the overall survival of patients in the validation set.


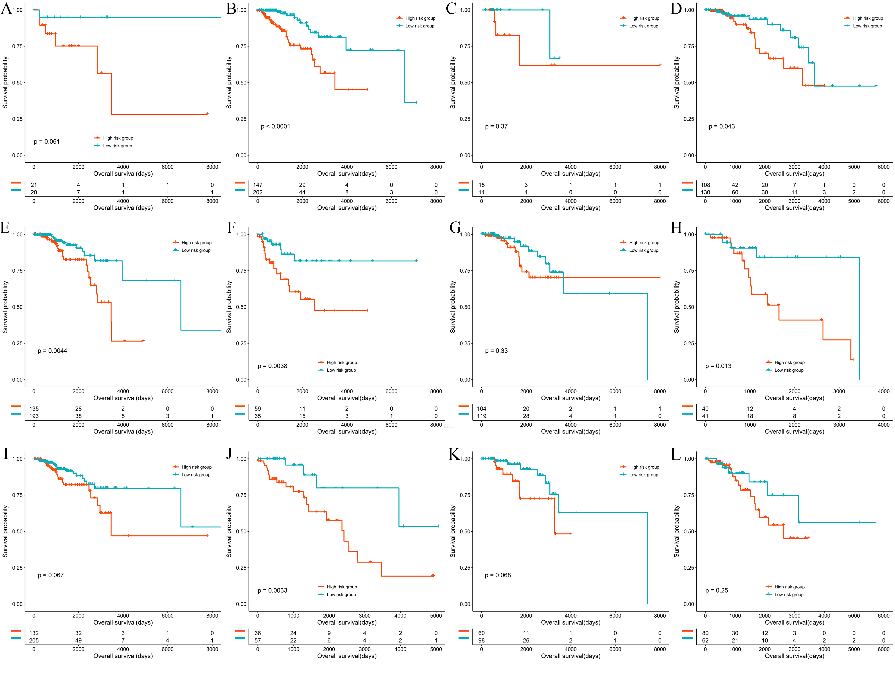


**Supplementary figure 3** Kaplan-Meier curve analysis on the overall survival in different subgroups. (A) Triple negative breast cancer in the discovery set; (B) Non-triple negative breast cancer in the validation set; (C) Triple negative breast cancer in the discovery set; (D) Non-triple negative breast cancer in the validation set; (E) Early stage breast cancer in the discovery set; (F) Advanced stage BC in the discovery set. (G) Early stage breast cancer in the validation set; (H) Advanced stage BC in the validation set. (I) Patients younger than 65 years in the discovery set; (J) Patients older than 65 years in the discovery set; (K) Patients younger than 65 years in the validation set; (L) Patients older than 65 years in the validation set;


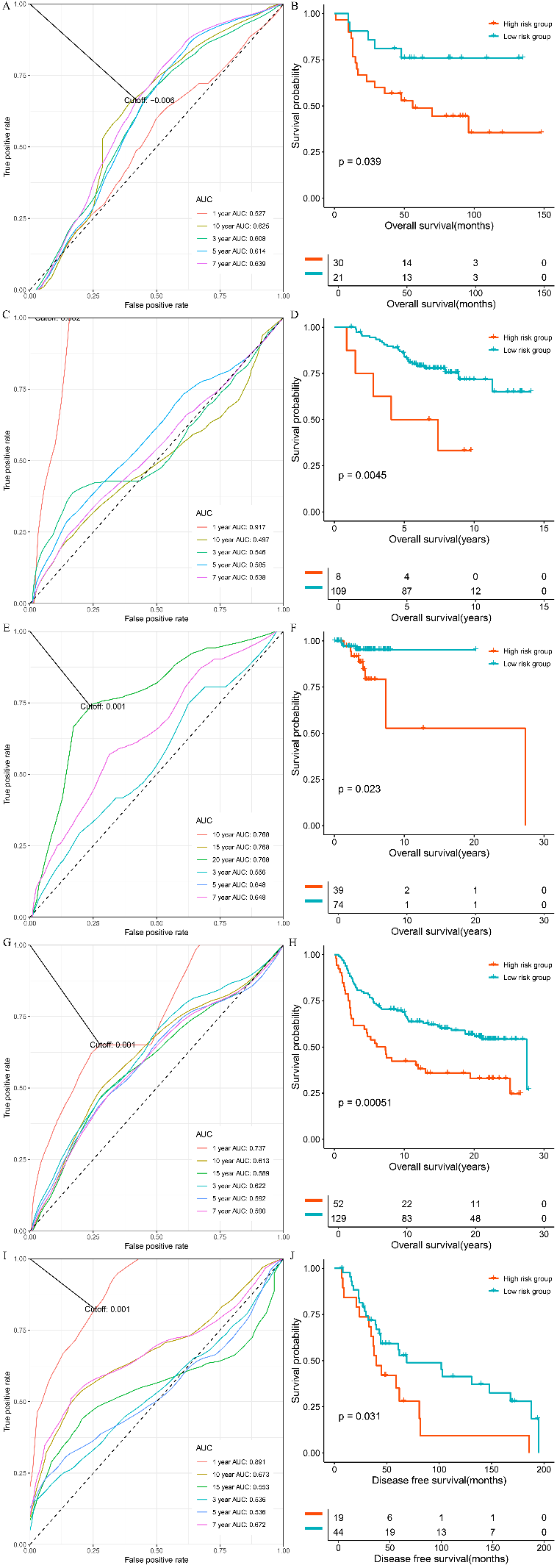


**Supplementary figure 4** The prognostic value of the multi CpG methylation panel in five independent validation cohort. (A) Time- dependent ROC analysis for predicting the OS of patients in GSE37754. (B) KM curves on the OS of patients in GSE37754. (C) Time- dependent ROC analysis for predicting the OS of patients in GSE72245. (D) KM curves on the OS of patients in GSE72245. (E) Time- dependent ROC analysis for predicting the OS of patients in GSE72251. (F) KM curves on the OS of patients in GSE72251. (G)Time- dependent ROC analysis for predicting the OS of patients in GSE75067. (H) KM curves on the OS of patients in GSE75067. (I) Time- dependent ROC analysis for predicting the OS of patients in GSE78754. (J) KM curves on the OS of patients in GSE78754.


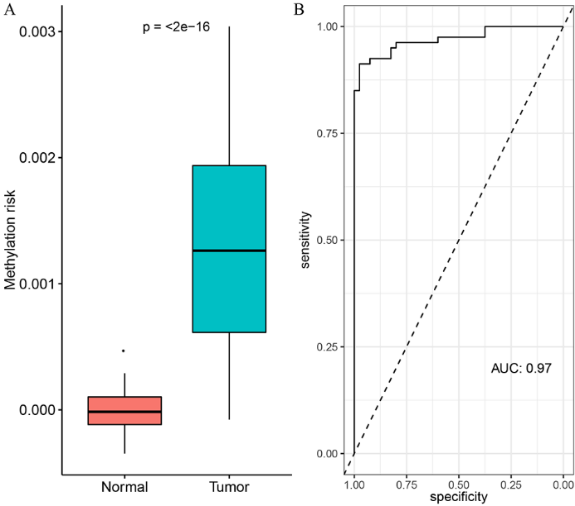


**Supplementary figure 5** The diagnostic performance of the multi CpG methylation panel in GSE66695. (A) The methylation risk of breast cancer patients and normal controls. (B) ROC curve for the classification of the multi CpG methylation panel.


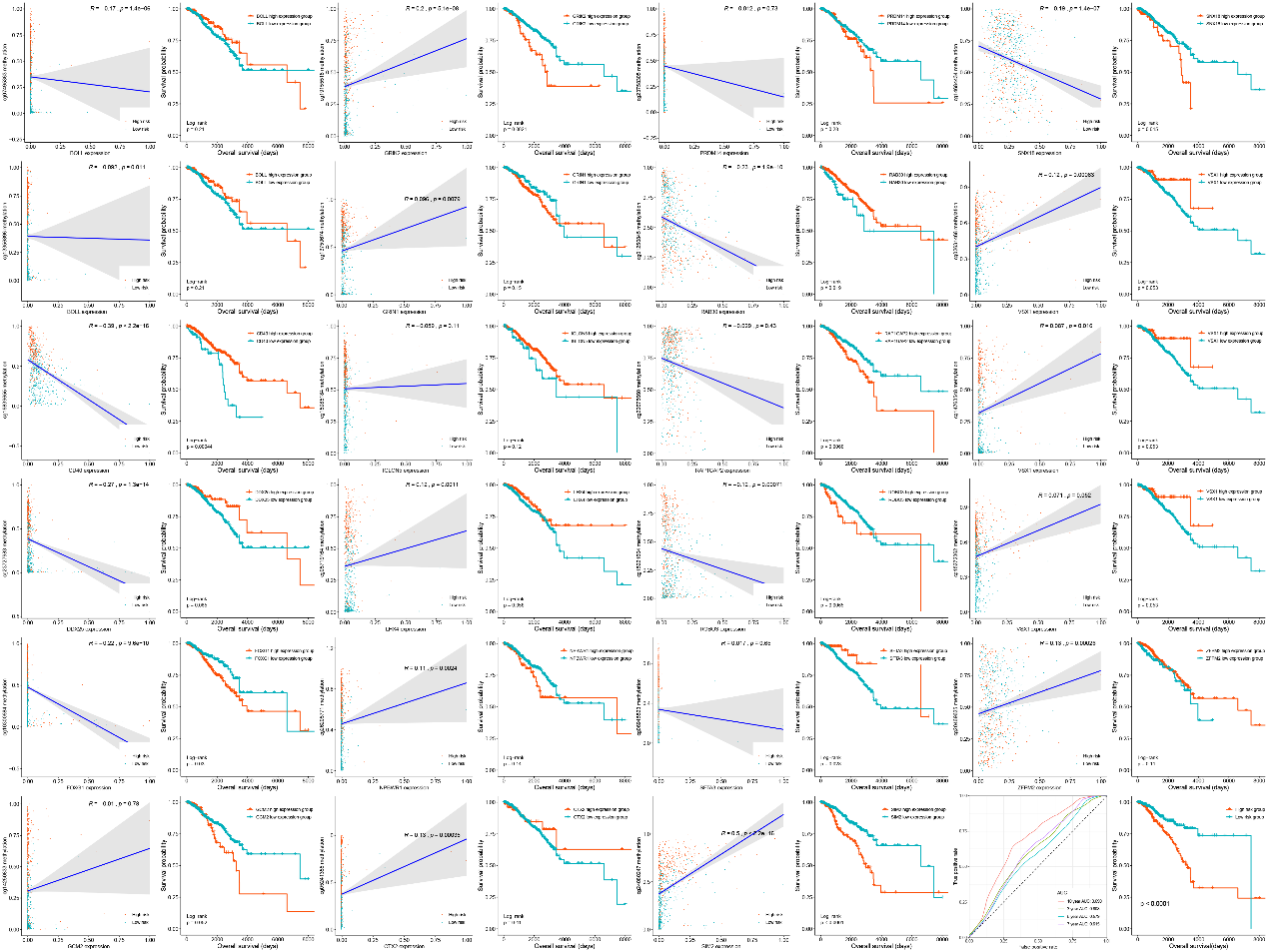


**Supplementary figure 6** The correlations between the methylation levels of CpG sites and their corresponding mRNAs and the survival relevance of these mRNA on the overall survival breast cancer patients.


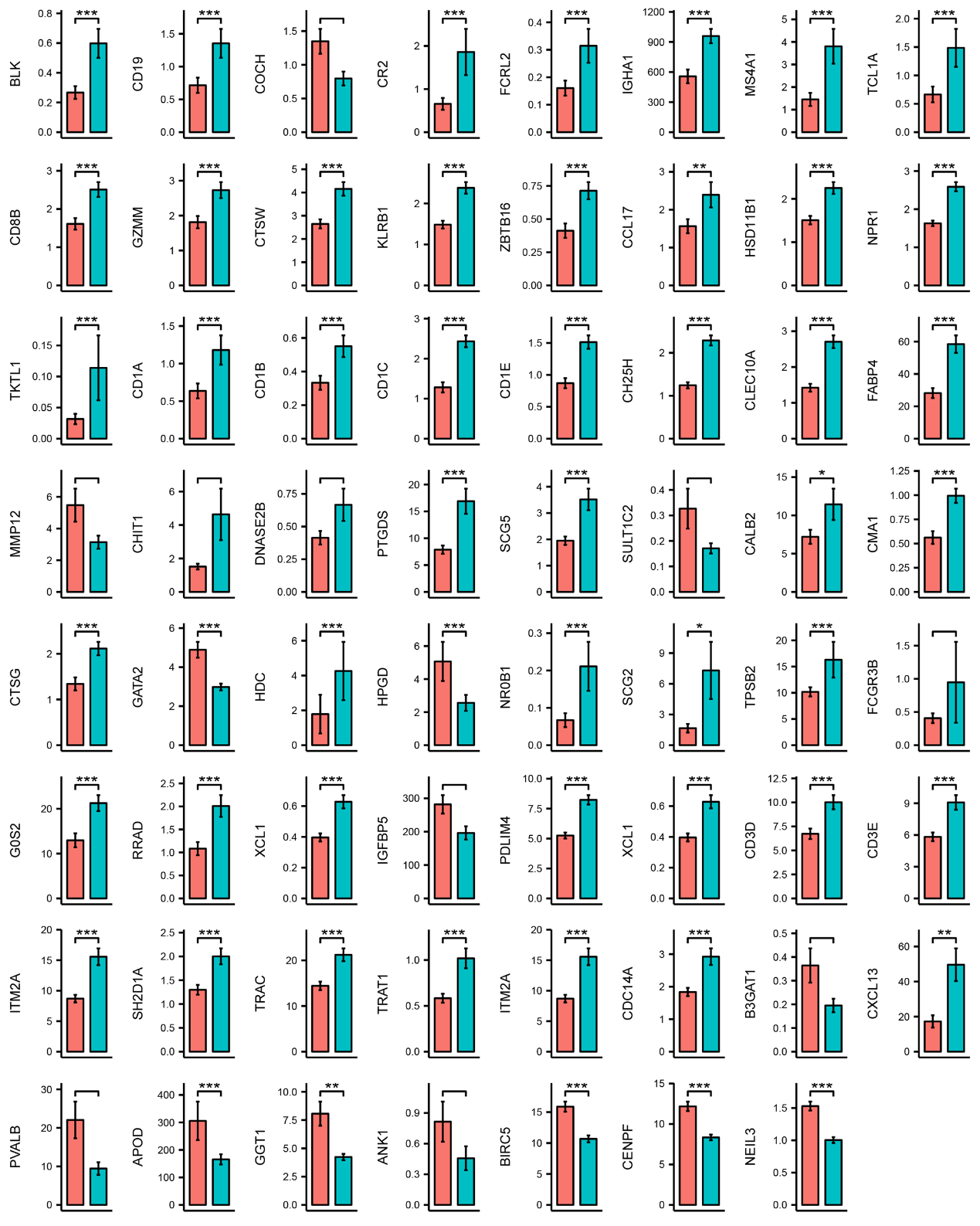


**Supplementary figure 7** The expression levels of genes specific for immune cells and differently expressed between the low-risk group and high-risk group. Notes: genes for B cells :BLK, CD19, COCH, CR2, FCRL2, IGHA1, MS4A1, and TCL1A; Genes for CD8^+^ T cells: CD8B and GZMM; genes for cytotoxic cells: CTSW, KLRB1, and ZBTB16; genes for DCs: CCL17, HSD11B1, and NPR1; gene for eosinophils: TKTL1; genes for iDCs: CD1A, CD1B, CD1C, CD1E, CH25H, CLEC10A, FABP4, and MMP12; genes for macrophages: CHIT1, DNASE2B, PTGDS, SCG5, and SULT1C2; genes for mast cells: CALB2, CMA1, CTSG, GATA2, HDC, HPGD, NR0B1, SCG2, and TPSB2; genes for neutrophils: FCGR3B, and G0S2; genes for NK CD56bright cells: RRAD, and XCL1; genes for NK cells: IGFBP5, PDLIM4, and XCL1; genes for T cells CD3D, CD3E, ITM2A, SH2D1A, TRAC, and TRAT1; gene for T helper cells: ITM2A; gene for Tcm cells: CDC14A; genes for Tfh cells: B3GAT1, CXCL13, and PVALB; genes for Th1 cells: APOD, and GGT1; genes for Th2 cells: ANK1, BIRC5, CENPF, and NEIL3.

**Supplementary table 1** Characteristics of patients in the discovery set

| Characteristics | **High risk** | **Low risk** | **Overall** |
| --- | --- | --- | --- |
|  | **(n=198)** | **(n=262)** | **(n=460)** |
| **Age (years)~** Median [Min, Max] | | |  |
|  | 60.0 [27.0, 90.0] | 54.0 [26.0, 88.0] | 57.0 [26.0, 90.0] |
| **Pathologic stage** |  |  |  |
| Stage I | 9 (4.5%) | 29 (11.1%) | 38 (8.3%) |
| Stage IA | 10 (5.1%) | 26 (9.9%) | 36 (7.8%) |
| Stage IB | 0 (0%) | 3 (1.1%) | 3 (0.7%) |
| Stage II | 3 (1.5%) | 2 (0.8%) | 5 (1.1%) |
| Stage IIA | 66 (33.3%) | 76 (29.0%) | 142 (30.9%) |
| Stage IIB | 47 (23.7%) | 57 (21.8%) | 104 (22.6%) |
| Stage III | 0 (0%) | 1 (0.4%) | 1 (0.2%) |
| Stage IIIA | 32 (16.2%) | 35 (13.4%) | 67 (14.6%) |
| Stage IIIB | 6 (3.0%) | 6 (2.3%) | 12 (2.6%) |
| Stage IIIC | 17 (8.6%) | 19 (7.3%) | 36 (7.8%) |
| Stage IV | 4 (2.0%) | 4 (1.5%) | 8 (1.7%) |
| **ER** |  |  |  |
| Negative | 54 (27.3%) | 50 (19.1%) | 104 (22.6%) |
| Positive | 136 (68.7%) | 194 (74.0%) | 330 (71.7%) |
| **PR** |  |  |  |
| Negative | 77 (38.9%) | 61 (23.3%) | 138 (30.0%) |
| Positive | 112 (56.6%) | 182 (69.5%) | 294 (63.9%) |
| **HER2** |  |  |  |
| Negative | 93 (47.0%) | 144 (55.0%) | 237 (51.5%) |
| Positive | 28 (14.1%) | 7 (2.7%) | 35 (7.6%) |

**Supplementary table 2** Characteristics of patients in the validation set

| Characteristics | **High risk** | **Low risk** | **Overall** |
| --- | --- | --- | --- |
|  | **(n=144)** | **(n=161)** | **(n=305)** |
| **Age (years)~** Median [Min, Max] | |  |  |
|  | 62.0 [29.0, 88.0] | 55.0 [26.0, 88.0] | 59.0 [26.0, 88.0] |
| **Pathologic stage** |  |  |  |
| Stage I | 6 (4.2%) | 14 (8.7%) | 20 (6.6%) |
| Stage IA | 12 (8.3%) | 13 (8.1%) | 25 (8.2%) |
| Stage IB | 1 (0.7%) | 1 (0.6%) | 2 (0.7%) |
| Stage II | 1 (0.7%) | 0 (0%) | 1 (0.3%) |
| Stage IIA | 50 (34.7%) | 45 (28.0%) | 95 (31.1%) |
| Stage IIB | 34 (23.6%) | 45 (28.0%) | 79 (25.9%) |
| Stage III | 1 (0.7%) | 0 (0%) | 1 (0.3%) |
| Stage IIIA | 26 (18.1%) | 30 (18.6%) | 56 (18.4%) |
| Stage IIIB | 4 (2.8%) | 1 (0.6%) | 5 (1.6%) |
| Stage IIIC | 7 (4.9%) | 8 (5.0%) | 15 (4.9%) |
| Stage IV | 1 (0.7%) | 2 (1.2%) | 3 (1.0%) |
| **ER** |  |  |  |
| Negative | 34 (23.6%) | 27 (16.8%) | 61 (20.0%) |
| Positive | 100 (69.4%) | 127 (78.9%) | 227 (74.4%) |
| **PR** |  |  |  |
| Negative | 52 (36.1%) | 40 (24.8%) | 92 (30.2%) |
| Positive | 82 (56.9%) | 113 (70.2%) | 195 (63.9%) |
| **HER2** |  |  |  |
| Negative | 70 (48.6%) | 91 (56.5%) | 161 (52.8%) |
| Positive | 16 (11.1%) | 4 (2.5%) | 20 (6.6%) |

**Supplementary table 3** Characteristics of patients in the GSE37754

| Characteristics | **High risk** | **Low risk** | **Overall** |
| --- | --- | --- | --- |
|  | **(n=32)** | **(n=29)** | **(n=61)** |
| **Age (years)~** Median [Min, Max] | | |  |
|  | 57.0 [30.0, 91.0] | 46.0 [34.0, 75.0] | 51.0 [30.0, 91.0] |
| **Node status** | |  |  |
| N0 | 19 (59.4%) | 14 (48.3%) | 33 (54.1%) |
| N1 | 11 (34.4%) | 12 (41.4%) | 23 (37.7%) |
| N2 | 2 (6.2%) | 2 (6.9%) | 4 (6.6%) |
| **Neoadjuvant therapy** | |  |  |
| No | 27 (84.4%) | 23 (79.3%) | 50 (82.0%) |
| Yes | 5 (15.6%) | 5 (17.2%) | 10 (16.4%) |
| **Hormone therapy** | |  |  |
| No | 18 (56.2%) | 17 (58.6%) | 35 (57.4%) |
| Yes | 12 (37.5%) | 10 (34.5%) | 22 (36.1%) |
| **Chemotherapy** | |  |  |
| No | 11 (34.4%) | 12 (41.4%) | 23 (37.7%) |
| Yes | 18 (56.2%) | 12 (41.4%) | 30 (49.2%) |

**Supplementary table 4** Characteristics of patients in the GSE72251

| Characteristics | **High risk** | **Low risk** | **Overall** |
| --- | --- | --- | --- |
|  | **(n=40)** | **(n=79)** | **(n=119)** |
| **Age (years)~** Median [Min, Max] | |  |  |
|  | 59.3 [23.4, 82.8] | 57.6 [30.2, 91.4] | 58.4 [23.4, 91.4] |
| **Subtype** |  |  |  |
| Basal | 8 (20.0%) | 26 (32.9%) | 34 (28.6%) |
| HER2 | 18 (45.0%) | 7 (8.9%) | 25 (21.0%) |
| LumA | 2 (5.0%) | 25 (31.6%) | 27 (22.7%) |
| LumB | 11 (27.5%) | 20 (25.3%) | 31 (26.1%) |
| **Grade** |  |  |  |
| 1 | 2 (5.0%) | 26 (32.9%) | 28 (23.5%) |
| 2 | 2 (5.0%) | 3 (3.8%) | 5 (4.2%) |
| 3 | 36 (90.0%) | 49 (62.0%) | 85 (71.4%) |
| **Size (cm)~** Mean (SD) | | |  |
|  | 3.09 (3.44) | 2.16 (1.82) | 2.48 (2.51) |
| **Node status** | |  |  |
| Negative | 18 (45.0%) | 50 (63.3%) | 68 (57.1%) |
| Positive | 22 (55.0%) | 26 (32.9%) | 48 (40.3%) |
| **ER** |  |  |  |
| Negative | 20 (50.0%) | 29 (36.7%) | 49 (41.2%) |
| Positive | 20 (50.0%) | 50 (63.3%) | 70 (58.8%) |
| **PR** |  |  |  |
| Negative | 28 (70.0%) | 36 (45.6%) | 64 (53.8%) |
| Positive | 12 (30.0%) | 42 (53.2%) | 54 (45.4%) |
| **HER2** |  |  |  |
| Negative | 22 (55.0%) | 72 (91.1%) | 94 (79.0%) |
| Positive | 18 (45.0%) | 7 (8.9%) | 25 (21.0%) |
| **Ki67~** Mean (SD) | |  |  |
|  | 38.4 (24.1) | 33.3 (30.3) | 35.0 (28.4) |

Supplementary table 5 Characteristics of patients in the GSE72245

| Characteristics | **High risk** | **Low risk** | **Overall** |
| --- | --- | --- | --- |
|  | **(n=8)** | **(n=110)** | **(n=118)** |
| **Age (years)~** Median [Min, Max] | | |  |
|  | 54.7 [41.9, 74.2] | 53.9 [28.6, 82.1] | 54.0 [28.6, 82.1] |
| **Grade** |  |  |  |
| 1 | 1 (12.5%) | 24 (21.8%) | 25 (21.2%) |
| 2 | 0 (0%) | 9 (8.2%) | 9 (7.6%) |
| 3 | 7 (87.5%) | 77 (70.0%) | 84 (71.2%) |
| **Subtype** |  |  |  |
| Basal | 3 (37.5%) | 28 (25.5%) | 31 (26.3%) |
| HER2 | 2 (25.0%) | 28 (25.5%) | 30 (25.4%) |
| LumA | 1 (12.5%) | 24 (21.8%) | 25 (21.2%) |
| LumB | 2 (25.0%) | 30 (27.3%) | 32 (27.1%) |
| **Size_cm (cm)~** Mean (SD) | | | |
|  | 2.68 (0.870) | 2.60 (1.46) | 2.61 (1.42) |
| **Node status** |  |  |  |
| Negative | 4 (50.0%) | 60 (54.5%) | 64 (54.2%) |
| Positive | 4 (50.0%) | 50 (45.5%) | 54 (45.8%) |
| **ER** |  |  |  |
| Negative | 5 (62.5%) | 48 (43.6%) | 53 (44.9%) |
| Positive | 3 (37.5%) | 61 (55.5%) | 64 (54.2%) |
| **HER2** |  |  |  |
| Negative | 6 (75.0%) | 82 (74.5%) | 88 (74.6%) |
| Positive | 2 (25.0%) | 28 (25.5%) | 30 (25.4%) |

**Supplementary table 6** characteristics of patients in the GSE75067

| Characteristics | **High risk** | **Low risk** | **Overall** |
| --- | --- | --- | --- |
|  | **(n=52)** | **(n=129)** | **(n=181)** |
| **Age** **(years)~**Median [Min, Max] | | |  |
|  | 55.1 [35.1, 86.0] | 47.2 [26.8, 82.8] | 48.2 [26.8, 86.0] |
| **ER** |  |  |  |
| Negative | 28 (53.8%) | 46 (35.7%) | 74 (40.9%) |
| Positive | 22 (42.3%) | 71 (55.0%) | 93 (51.4%) |
| **PR** |  |  |  |
| Negative | 32 (61.5%) | 47 (36.4%) | 79 (43.6%) |
| Positive | 17 (32.7%) | 68 (52.7%) | 85 (47.0%) |
| **Size~** Mean (SD) | | | |
|  | 32.1 (22.0) | 22.5 (9.98) | 25.2 (15.0) |
| **node status** | |  |  |
| Negative | 20 (38.5%) | 71 (55.0%) | 91 (50.3%) |
| Positive | 20 (38.5%) | 36 (27.9%) | 56 (30.9%) |
| **Grade** |  |  |  |
| 1 | 2 (3.8%) | 19 (14.7%) | 21 (11.6%) |
| 2 | 13 (25.0%) | 34 (26.4%) | 47 (26.0%) |
| 3 | 30 (57.7%) | 54 (41.9%) | 84 (46.4%) |
| **tumor type** | |  |  |
| ductal | 33 (63.5%) | 84 (65.1%) | 117 (64.6%) |
| lobular | 1 (1.9%) | 5 (3.9%) | 6 (3.3%) |
| medullary | 1 (1.9%) | 2 (1.6%) | 3 (1.7%) |
| mixed type | 1 (1.9%) | 8 (6.2%) | 9 (5.0%) |
| noninvasive | 0 (0%) | 1 (0.8%) | 1 (0.6%) |
| special types | 0 (0%) | 4 (3.1%) | 4 (2.2%) |
| tubular | 2 (3.8%) | 2 (1.6%) | 4 (2.2%) |

**Supplementary table 7** CpG sites that were significantly related with the overall survival of breast cancer patients

|  | HR | LCI | UCI | P Value | Gene symbol |
| --- | --- | --- | --- | --- | --- |
| cg06945523 | 4.138632 | 2.062073 | 8.306336 | 6.44E-05 | SFTA3 |
| cg15239627 | 0.188406 | 0.081071 | 0.437853 | 0.000105 | C7orf58 |
| cg01751245 | 0.086244 | 0.024857 | 0.299237 | 0.000113 | SPRED2 |
| cg09884146 | 0.05563 | 0.012165 | 0.254406 | 0.000196 | SPRED2 |
| cg02631468 | 5.984239 | 2.321617 | 15.42507 | 0.000213 | VSX1 |
| cg14565781 | 0.16898 | 0.063064 | 0.45278 | 0.000407 | BTBD19 |
| cg05241355 | 3.772004 | 1.806291 | 7.876922 | 0.00041 | OTX2 |
| cg03686593 | 2.93015 | 1.613217 | 5.322148 | 0.000415 | FAM38B |
| cg25160286 | 5.087669 | 1.996598 | 12.96424 | 0.000652 | EVX1 |
| cg01250845 | 0.217413 | 0.089199 | 0.529922 | 0.000788 | RAB30 |
| cg19418318 | 0.254896 | 0.114456 | 0.567657 | 0.00082 | MYO9B |
| cg00991994 | 0.203229 | 0.079408 | 0.520126 | 0.00089 | C3orf26 |
| cg02919712 | 0.148364 | 0.047513 | 0.463275 | 0.001022 | DEPDC6 |
| cg15269394 | 0.221687 | 0.090178 | 0.544982 | 0.001028 | ATP6V0A1 |
| cg24080247 | 3.806521 | 1.712243 | 8.462352 | 0.00104 | SIM2 |
| cg02493602 | 4.200259 | 1.767302 | 9.982549 | 0.001157 | ME3 |
| cg13569051 | 0.191491 | 0.070378 | 0.521024 | 0.00121 | GSN |
| cg23758305 | 4.852338 | 1.863436 | 12.63536 | 0.001218 | PRDM14 |
| cg16703956 | 4.260618 | 1.758785 | 10.32126 | 0.001324 | SLC6A3 |
| cg05940984 | 4.672097 | 1.819646 | 11.99601 | 0.001354 | WT1 |
| cg14763548 | 3.008141 | 1.52938 | 5.916721 | 0.001418 | VSX1 |
| cg13820205 | 0.084371 | 0.018456 | 0.385698 | 0.00143 | PRRX2 |
| cg20469625 | 0.246876 | 0.103354 | 0.589697 | 0.001639 | ZFPM2 |
| cg05719164 | 3.053447 | 1.522016 | 6.125779 | 0.001676 | LHX4 |
| cg00348598 | 0.228004 | 0.090636 | 0.573565 | 0.001684 | BMPER |
| cg09524907 | 4.016893 | 1.668243 | 9.672108 | 0.001926 | XKR4 |
| cg24595510 | 2.99602 | 1.495019 | 6.004027 | 0.001976 | SCRT2 |
| cg23807890 | 3.51387 | 1.58204 | 7.804655 | 0.002025 | WDR69 |
| cg02062480 | 3.760687 | 1.620448 | 8.727692 | 0.002044 | NR2E1 |
| cg19839655 | 4.608207 | 1.744714 | 12.17138 | 0.002048 | CD40 |
| cg15603568 | 3.276241 | 1.539881 | 6.97051 | 0.002065 | GRIA4 |
| cg08467103 | 0.103326 | 0.024373 | 0.438041 | 0.00207 | SPRED2 |
| cg25161912 | 0.117858 | 0.029947 | 0.463835 | 0.002221 | HMGA2 |
| cg15272362 | 4.831532 | 1.748089 | 13.35384 | 0.002392 | VSX1 |
| cg22675660 | 0.280305 | 0.12326 | 0.637438 | 0.002412 | RAP1GAP2 |
| cg27039118 | 0.110002 | 0.026331 | 0.459552 | 0.00248 | TRPS1 |
| cg13209481 | 6.823121 | 1.955412 | 23.80828 | 0.002598 | SFTA3 |
| cg02446647 | 4.053682 | 1.629948 | 10.08151 | 0.002604 | EYA4 |
| cg05863502 | 5.796772 | 1.845674 | 18.20613 | 0.002617 | CACNA1B |
| cg01105356 | 0.20446 | 0.072559 | 0.576137 | 0.002672 | CIITA |
| cg01995743 | 3.28559 | 1.505587 | 7.170026 | 0.002811 | SLC35F1 |
| cg19155518 | 3.095938 | 1.472049 | 6.51122 | 0.002889 | GRIK2 |
| cg07495363 | 3.025362 | 1.459288 | 6.272111 | 0.00292 | BOLL |
| cg26205771 | 3.340938 | 1.508675 | 7.398457 | 0.002942 | NPBWR1 |
| cg14250833 | 2.608869 | 1.379426 | 4.934078 | 0.003185 | GCM2 |
| cg03326762 | 4.287626 | 1.627362 | 11.29665 | 0.003228 | LHX8 |
| cg10210510 | 0.257362 | 0.104251 | 0.635344 | 0.003243 | COL9A2 |
| cg17887364 | 0.183681 | 0.059399 | 0.567999 | 0.003261 | EIF4EBP1 |
| cg16793866 | 3.7544 | 1.550505 | 9.090925 | 0.003368 | TMEM90B |
| cg02650401 | 5.391368 | 1.740874 | 16.6967 | 0.003487 | SOX2OT |
| cg23727983 | 2.932918 | 1.42368 | 6.042095 | 0.003524 | DDX25 |
| cg10300684 | 3.387822 | 1.491137 | 7.697037 | 0.003566 | FOXG1 |
| cg15985184 | 4.153814 | 1.592595 | 10.834 | 0.003598 | IGLON5 |
| cg09829319 | 3.058768 | 1.439792 | 6.498203 | 0.003637 | GCM2 |
| cg11413039 | 3.929632 | 1.555669 | 9.926281 | 0.003796 | PUS3 |
| cg19229692 | 0.204642 | 0.069652 | 0.601252 | 0.003912 | PLXNA4 |
| cg09734791 | 3.133516 | 1.440848 | 6.814682 | 0.00396 | MSC |
| cg15221604 | 3.161234 | 1.442745 | 6.926658 | 0.00403 | ROBO3 |
| cg00553149 | 5.641018 | 1.734391 | 18.34712 | 0.00404 | STAG3 |
| cg03184290 | 4.075134 | 1.558687 | 10.6543 | 0.004168 | PITX2 |
| cg01834022 | 2.738041 | 1.372712 | 5.461354 | 0.004247 | EVC2 |
| cg06327814 | 0.215396 | 0.075184 | 0.617096 | 0.004252 | C7orf53 |
| cg14502484 | 4.280189 | 1.576673 | 11.61942 | 0.004323 | SLC6A3 |
| cg14684434 | 0.264853 | 0.105893 | 0.662433 | 0.004505 | SNX18 |
| cg04456932 | 0.310021 | 0.137972 | 0.696615 | 0.00458 | ZNF783 |
| cg16851417 | 3.640793 | 1.486137 | 8.919351 | 0.004705 | SIM2 |
| cg13356896 | 2.55872 | 1.331094 | 4.918545 | 0.004837 | BOLL |
| cg13759674 | 2.900854 | 1.3815 | 6.09117 | 0.004896 | GRIN1 |

**Supplementary table 8** The coefficients of the candidate CpG islands

| CpG | Coefficient |
| --- | --- |
| cg00991994 | -6.99E-05 |
| cg01250845 | -0.000171859 |
| cg02631468 | 0.000120587 |
| cg02919712 | -3.30E-05 |
| cg03686593 | 0.000678749 |
| cg05241355 | 0.000186204 |
| cg05719164 | 5.45E-05 |
| cg06327814 | -2.95E-05 |
| cg06945523 | 0.000127575 |
| cg07495363 | 0.000135586 |
| cg10300684 | 0.000187188 |
| cg13356896 | 0.00069376 |
| cg13759674 | 0.000296361 |
| cg14250833 | 0.000126587 |
| cg14684434 | -1.46E-05 |
| cg14763548 | 0.000222171 |
| cg15221604 | 0.000200218 |
| cg15272362 | 2.94E-05 |
| cg15985184 | 9.07E-05 |
| cg19155518 | 0.000245764 |
| cg19839655 | 3.45E-05 |
| cg20469625 | -0.000223433 |
| cg22675660 | -0.000229579 |
| cg23727983 | 0.000200793 |
| cg23758305 | 0.000264712 |
| cg23807890 | 7.00E-05 |
| cg24080247 | 0.00014155 |
| cg26205771 | 0.000180752 |

**Supplementary table 9** Cox proportional hazards regression model of patients in the discovery set.

| Characteristics | Univariate analysis | | | |  | Multivariable analysis | | | | Reference |
| --- | --- | --- | --- | --- | --- | --- | --- | --- | --- | --- |
|  | HR | LCI | UCI | P value |  | HR | LCI | UCI | P value |  |
| Group | 0.366 | 0.217 | 0.619 | <0.0001 |  | 0.438 | 0.248 | 0.77 | 0.004 | High risk |
| Age | 1.041 | 1.02 | 1.061 | <0.0001 |  | 1.038 | 1.016 | 1.059 | 0.001 | / |
| ER | 0.685 | 0.39 | 1.203 | 0.188 |  | / | | | | Negative |
| PR | 0.734 | 0.426 | 1.266 | 0.267 |  | / | | | | Negative |
| Pathological stage | 1.376 | 1.178 | 1.606 | <0.0001 |  | 1.35 | 1.16 | 1.571 | <0.0001 | / |
| HER2 | 0.425 | 0.102 | 1.769 | 0.24 |  | / | | | | Negative |

**Abbreviations:** HR, hazards ratio; LCI, lower limit of confidence interval; UCI, upper limit of confidence interval.

**Supplementary table 10** Cox proportional hazards regression model of patients in the validation set

| Characteristics | Univariate analysis | | | |  | Multivariable analysis | | | | Reference |
| --- | --- | --- | --- | --- | --- | --- | --- | --- | --- | --- |
|  | HR | LCI | UCI | P value |  | HR | LCI | UCI | P value |  |
| Group | 0.476 | 0.251 | 0.901 | 0.023 |  | 0.558 | 0.282 | 1.105 | 0.094 | High risk |
| Age | 1.032 | 1.005 | 1.06 | 0.019 |  | 1.039 | 1.008 | 1.071 | 0.013 | / |
| ER | 0.655 | 0.327 | 1.312 | 0.233 |  | / | | | | Negative |
| PR | 0.891 | 0.458 | 1.735 | 0.735 |  | / | | | | Negative |
| Pathologic stage | 1.669 | 1.349 | 2.066 | <0.0001 |  | 1.755 | 1.418 | 2.171 | <0.0001 | / |
| HER2 | 1.984 | 0.647 | 6.088 | 0.231 |  | / | | | | Negative |

**Abbreviations:** HR, hazards ratio; LCI, lower limit of confidence interval; UCI, upper limit of confidence interval.

**Supplementary table 11** Cox proportional hazards regression model of patients in GSE37754

| Characteristics | Univariate analysis | | | |  | Multivariable analysis | | | | Reference |
| --- | --- | --- | --- | --- | --- | --- | --- | --- | --- | --- |
|  | HR | LCI | UCI | P value |  | HR | LCI | UCI | P value |  |
| Group | 0.364 | 0.134 | 0.99 | 0.048 |  | 0.299 | 0.094 | 0.95 | 0.041 | High risk |
| Age | 1.433 | 0.937 | 2.194 | 0.097 |  | 0.914 | 0.471 | 1.771 | 0.789 | / |
| Node status N1 | 1.986 | 0.797 | 4.949 | 0.141 |  | 5.455 | 1.762 | 16.888 | 0.003 | N0 |
| Node status N2 | 3.658 | 0.974 | 13.734 | 0.055 |  | 3.709 | 0.845 | 16.29 | 0.083 |  |
| Neoadjuvant therapy | 7.543 | 3.048 | 18.664 | <0.0001 |  | 15.904 | 4.145 | 61.027 | <0.0001 | No |
| Chemotherapy | 0.837 | 0.361 | 1.942 | 0.679 |  | 0.213 | 0.052 | 0.871 | 0.031 | No |
| Hormone therapy | 0.53 | 0.204 | 1.374 | 0.191 |  | 0.135 | 0.035 | 0.516 | 0.003 | No |

**Abbreviations:** HR, hazards ratio; LCI, lower limit of confidence interval; UCI, upper limit of confidence interval.

**Supplementary table 12** Cox proportional hazards regression model of patients in GSE72245

| Characteristics | Univariate analysis | | | |  | Multivariable analysis | | | | Reference |
| --- | --- | --- | --- | --- | --- | --- | --- | --- | --- | --- |
|  | HR | LCI | UCI | P value |  | HR | LCI | UCI | P value |  |
| Group | 0.272 | 0.104 | 0.714 | 0.008 |  | 0.32 | 0.115 | 0.889 | 0.029 | High risk |
| subtype HER2 | 1.166 | 0.505 | 2.692 | 0.72 |  | 1.518 | 0.588 | 3.92 | 0.388 | Basal |
| Subtype LumA | 0.185 | 0.041 | 0.836 | 0.028 |  | 0.752 | 0.02 | 28.221 | 0.877 |  |
| Subtype LumB | 0.555 | 0.214 | 1.435 | 0.224 |  | 1.122 | 0.227 | 5.546 | 0.887 |  |
| Size | 1.308 | 1.018 | 1.681 | 0.036 |  | 1.265 | 0.923 | 1.734 | 0.143 | / |
| Node status | 1.618 | 0.795 | 3.29 | 0.184 |  | / | | | | Negative |
| Age | 1.615 | 1.101 | 2.366 | 0.014 |  | 1.748 | 1.183 | 2.583 | 0.005 | / |
| ER | 0.406 | 0.194 | 0.849 | 0.017 |  | 0.526 | 0.131 | 2.118 | 0.366 | Negative |
| Grade | 1.834 | 1.081 | 3.113 | 0.025 |  | 1.322 | 0.385 | 4.544 | 0.658 | / |
| HER2 | 2.005 | 0.96 | 4.19 | 0.064 |  | / | | | | Negative |

**Abbreviations:** HR, hazards ratio; LCI, lower limit of confidence interval; UCI, upper limit of confidence interval.

**Supplementary table 13** Cox proportional hazards regression model of patients in GSE72251

| Characteristics | Univariate analysis | | | |  | Multivariable analysis | | | | Reference |
| --- | --- | --- | --- | --- | --- | --- | --- | --- | --- | --- |
|  | HR | LCI | UCI | P value |  | HR | LCI | UCI | P value |  |
| Group | 0.237 | 0.061 | 0.918 | 0.037 |  | 1.789 | 0.878 | 3.646 | 0.109 | High risk |
| Subtype HER2 | 0.357 | 0.072 | 1.785 | 0.21 |  | / | | | | Basal |
| Subtype LumA | 0 | 0 | Inf | 0.998 |  |  |  |  |  |  |
| Subtype LumB | 0.281 | 0.055 | 1.433 | 0.127 |  |  |  |  |  |  |
| Grade | 1.731 | 0.702 | 4.267 | 0.234 |  | / | | | | / |
| Size | 1.192 | 0.842 | 1.689 | 0.322 |  | / | | | | / |
| Node status | 5.269 | 1.119 | 24.814 | 0.036 |  | 3.905 | 0.81 | 18.836 | 0.09 | Negative |
| PR | 0 | 0 | Inf | 0.998 |  | / | | | | Negative |
| ER | 0.154 | 0.032 | 0.737 | 0.019 |  | 0.197 | 0.041 | 0.951 | 0.043 | Negative |
| Age | 0.938 | 0.5 | 1.758 | 0.841 |  | / | | | | / |
| Ki67 | 1.469 | 0.816 | 2.645 | 0.2 |  | / | | | | / |
| HER2 | 0.864 | 0.183 | 4.073 | 0.853 |  | / | | | | Negative |

**Abbreviations:** HR, hazards ratio; LCI, lower limit of confidence interval; UCI, upper limit of confidence interval.

**Supplementary table 14** Cox proportional hazards regression model of patients in GSE75067

| Characteristics | Univariate analysis | | | |  | Multivariable analysis | | | | Reference |
| --- | --- | --- | --- | --- | --- | --- | --- | --- | --- | --- |
|  | HR | LCI | UCI | P value |  | HR | LCI | UCI | P value |  |
| Group | 0.478 | 0.312 | 0.732 | 0.001 |  | 0.565 | 0.344 | 0.929 | 0.025 | High risk |
| ER | 0.707 | 0.458 | 1.091 | 0.117 |  | / | | | | Negative |
| PR | 0.54 | 0.349 | 0.836 | 0.006 |  | 0.712 | 0.401 | 1.264 | 0.246 | Negative |
| Size | 1.649 | 1.376 | 1.977 | 0 |  | / | | | | / |
| Node status | 4.004 | 2.457 | 6.525 | 0 |  | / | | | | Negative |
| grade | 1.347 | 1.054 | 1.722 | 0.017 |  | 1.166 | 0.854 | 1.593 | 0.334 | / |
| Age | 1.232 | 0.994 | 1.526 | 0.057 |  | 1.194 | 0.94 | 1.518 | 0.147 | / |
| Subtype lobular | 1.091 | 0.342 | 3.482 | 0.884 |  |  | / |  |  | / |
| Subtype medullary | 0.541 | 0.075 | 3.928 | 0.544 |  |  |  |  |  |  |
| Subtype mixed type | 0.169 | 0.023 | 1.222 | 0.078 |  |  |  |  |  |  |
| Subtype noninvasive | 0 | 0 | Inf | 0.997 |  |  |  |  |  |  |
| Subtype special types | 0.423 | 0.059 | 3.057 | 0.394 |  |  |  |  |  |  |
| Subtype tubular | 0.433 | 0.06 | 3.125 | 0.406 |  |  |  |  |  |  |

**Abbreviations:** HR, hazards ratio; LCI, lower limit of confidence interval; UCI, upper limit of confidence interval.

**Supplementary table 15** Cox proportional hazards regression model of patients in GSE78754

| Characteristics | Univariate analysis | | | |  | Multivariable analysis | | | | Reference |
| --- | --- | --- | --- | --- | --- | --- | --- | --- | --- | --- |
|  | HR | LCI | UCI | P value |  | HR | LCI | UCI | P value |  |
| Group | 0.501 | 0.264 | 0.951 | 0.034 |  | 0.496 | 0.26 | 0.943 | 0.033 | High risk |
| Age | 0.999 | 0.976 | 1.021 | 0.902 |  | 0.996 | 0.973 | 1.02 | 0.742 |  |

**Abbreviations:** HR, hazards ratio; LCI, lower limit of confidence interval; UCI, upper limit of confidence interval.
